# Disrupted macrophage autophagy as a driver of cell death and LPS-induced lethal shock in systemic inflammation

**DOI:** 10.3389/fimmu.2025.1610033

**Published:** 2025-10-23

**Authors:** Tarek Chekroune, Sandra Carignon, Meriem Taleb, Florence Savigny, Stéphanie Rose, Isabelle Maillet, Catherine Mura

**Affiliations:** ^1^ Immuno NEuro Modulation (INEM), UMR 7355 CNRS, Orléans, France; ^2^ University of Orléans, Orléans, France

**Keywords:** SIRS, autophagy, macrophage, iron, ROS, cell death

## Abstract

Systemic inflammatory response syndrome (SIRS) can be primed by infectious or non-infectious stimuli and may progress to life-threatening organ failure. An altered balance between pro- and anti-inflammatory response is commonly observed in SIRS, yet the core molecular events driving severe SIRS remain poorly defined. Moreover, the roles of macrophages and autophagy in SIRS have been pointed out. Here a high susceptibility to LPS-induced lethal shock in mice deficient in autophagy in myeloid cells (*Atg5^f/f^LysM-cre^+^*) following a single dose of 0.5 mg/kg (60% mortality vs. 0% in wild type after 24h) was observed. Using a very low dose of LPS (0.1 mg/kg), *Atg5^f/f^LysM-cre^+^* mice showed rapid tissue injury, notably in the liver and spleen, accompanied by an altered macrophage phenotype. Macrophages in the spleen and the liver appeared swollen and showed a loss of cellular content, including iron. In contrast, hepatocytes in *Atg5^f/f^LysM-cre^+^* mice accumulated more iron, which was associated with elevated reactive oxygen species levels compared to wild-type mice. Notably, the livers of LPS-treated *Atg5^f/f^LysM-cre^+^* mice exhibited increased ferroptotic and apoptotic cell death and extensive pyroptosis in both the spleen and liver. Flow cytometric analysis, immunofluorescence, and RNA sequencing supported the marked pro-inflammatory phenotype of macrophages in LPS-treated *Atg5^f/f^LysM-cre^+^* mice. In conclusion, during LPS-induced inflammation, autophagy deficiency in myeloid cells profoundly alters macrophage phenotype, disrupts iron trafficking, and promotes tissue injury through multiple forms of cell death.

## Introduction

The inflammatory response may be exaggerated or prolonged, potentially progressing to systemic inflammatory response syndrome (SIRS), which can be detrimental by causing multi-organ failure and respiratory distress syndrome, ultimately leading to death (1–5). SIRS has a heterogeneous spectrum of etiologies; it can be triggered by pathogens or non-infectious noxious stimuli (6), or it can accompany intestinal dysbiosis (7). SIRS is characterized by an initial, pronounced increase in the levels of multiple early pro-inflammatory mediators, including TNFα and IL-1β, primarily secreted by activated monocytes and macrophages, as well as IL-6, which is produced in response to tissue injury, thus resulting in a “cytokine storm”. Later, there is a sustained overproduction of the anti-inflammatory cytokine IL-10 (2, 3, 6). However, the primary molecular event driving the massive inflammatory response and tissue damage observed in SIRS is not yet precisely defined.

Macrophages are widely distributed throughout the body and exhibit remarkable plasticity (8, 9). Subsets of resident macrophages contribute to various tissue functions independently of immune-cell signaling, including organogenesis, tissue homeostasis, and acting as sentinels (8, 10). Indeed splenic macrophages, as well as Kupffer cells, are crucial for the clearance of senescent and damaged erythrocytes by phagocytosis as well as iron recycling via the iron exporter ferroportin (8). Macrophages also play a role in tissue homeostasis through the removal of cellular debris and apoptotic cells. Furthermore, macrophages are essential for mounting an appropriate inflammatory response, and excessive activation may be deleterious (2). Indeed macrophages are involved in initiating and controlling the immune response during the early stage. During the later stage, the clearance function of macrophages—through phagocytic and autophagic activities—is crucial for eliminating pathogens, stimulators, and apoptotic immune cells, thereby limiting the release of apoptotic cell antigens and autoantigens (8, 11–14). Additionally, the excessive apoptosis of macrophages and other immune cells increases sharply, leading to immune suppression and organ injury (11, 12), and antagonizing apoptosis of immune cells, including macrophages, rather than other inflammatory factors, significantly reduces organ damage in SIRS (12).

Autophagy affects macrophage functional immune properties. Indeed TLR-mediated inflammation induced autophagy, which degrades and thus modulates cytokine production, particularly IL-1β and TNfα, and autophagy has a negative role in inflammasome activation (11, 15–18). Conversely, inflammatory signals, including IL-1β and TNFα, can activate autophagy (11, 12). The autophagic machinery is also used for ingestion of extracellular structure through LC3-associated phagocytosis, a process that uses ATG proteins and is triggered by surface receptors such as TLRs (19, 20). Therefore, autophagy in macrophages contributes to the clearance of pathogens, antigen presentation, and efferocytosis (21). Uncontrolled macrophage autophagy could thus be responsible for the pathophysiological changes in SIRS.

In mice, LPS treatment is a common model to induce systemic inflammatory response; however, the molecular pathway that leads to the “cytokine storm”, morbidity, and mortality is poorly characterized. The murine response to lipopolysaccharide (LPS) exhibits a dose-dependent spectrum; a high dose of gram-negative bacterium LPS triggers the production of large amounts of pro-inflammatory cytokines and mediators and life-threatening conditions including hypotension and hypothermia, which rapidly lead to death (22). The role of autophagy in LPS endotoxemia was suggested by studies using mice deficient in autophagy. Mice deficient in Lc3b (*Map1Lc3b* ^-/-^), a protein involved in the autophagy pathway, treated with a single high dose of 12 mg LPS/kg exhibited high lethality with marked activation of caspase-1 activity (11). Mice deficient in autophagy specifically in macrophages (*Atg5^f/f^LysM-cre*) subjected either to an endotoxemic dose of LPS (chronic dose of 0.25 mg/kg/day/2 weeks) or a co-injection of LPS and galactosamine (0.1–700 mg/kg) showed acute liver injury, increased levels of proinflammatory cytokines, M1 polarization, and apoptotic cell death (23, 24). These observations clearly implicate autophagy in macrophages as a critical mechanism in LPS-induced shock and have prompted us to further investigate its contribution and the underlying molecular mechanisms of autophagy-mediated signaling involved in LPS-induced tissue damage.

## Materials and methods

### Animal model

*Atg5^f/f^* (*Atg5^tm1Myok^* 25) were bred with *Atg5^fl/fl^ LysMCre^+^* mice (mice carrying one allele of LysMCre; 26) to create myeloid lineage cell-specific Atg5 deletion (*Atg5^f/f^LysM-cre^+^*). Wild-type littermates, when available, were used as controls. The mice were housed under specific pathogen-free conditions and provided with a 12-h/12-h light/dark cycle in our animal facility at the Transgenose Institute (UAR44 TAAM CNRS, Orléans, France) in ventilated cages enriched with rodent homes and nesting papers. The animals had *ad libitum* access to chow and water. Mice, male and female, that were 12 to 15 weeks old (25–30 g) were used in this study. The mice were fed a standard commercial diet (R03 from Scientific Animal Food & Engineering; Augy, France) with a normal iron content of 250 mg iron/kg. All animal experiments complied with the French Government’s animal experiment regulations and were approved by the National Ethics Committee for Animal Experimentation (CLE CCO 2020-23557). Myeloid-specific gene deletion was confirmed by specific gene inactivation in bone-marrow-derived macrophages as well as in splenic and liver isolated macrophages, while Atg5 protein was expressed in total liver extract (**Supplementary Figure S1**) from *Atg5^f/f^ LysM-cre^+^* mice.

The mice were administered with a single intraperitoneal injection of 0.2 mL of a solution of LPS from *Escherichia coli* serotype 055:B5 (Sigma-Aldrich, St. Louis, MO, USA) at 0.5 or 0.1 mg/kg body weight. Sterile saline solution (0.9%) was used as vehicle control. A clinical follow-up after LPS injection to assess the severity of the illness was performed using a clinical scoring scale that included animal appearance (score 0–2), mobility (score 0–2), and fur ruffling (score 0–2), with a final kill score of 4.

### Hematology, serum, and iron parameters

The mice were anesthetized with isoflurane, and blood was collected by retroorbital venipuncture in EDTA-containing tubes for plasma or in serum separator tubes (BD Biosciences, Franklin Lakes, NJ, USA). Hematological parameters and leukocyte populations were determined by using an SCIL Vet abc Plus^+^ hematometer (SCIL Animal Care Company, Altorf, France). Aspartate aminotransferase (AST) and alanine aminotransferase (ALT) levels were determined by a colorimetric method (Biolabo SAS, Maizy, France). Serum iron parameters and nonheme tissue iron content were analyzed as described previously (27). Briefly, dried tissue samples were weighed and dissolved in 1:1 sulfuric acid/nitric acid at 90°C. After the addition of 1 mL of H_2_O, 25 µL of solution was reacted with ferrozine detection solution (10 mM ferrozine/32.6 mM L-ascorbic acid/50 mM Tris HCl pH 4), and the absorbance was then measured at 562 nm. A standard FeCl_3_ curve was used to calculate the iron concentration. Tissue iron content was reported as nanogram of iron per milligram of dried tissue.

### Flow cytometry cell sorting

Single-cell suspensions of the liver were obtained by enzymatic digestion with liberase (125 μg/mL; Roche Life Science, Penzberg, Germany) and DNase I (1 mg/mL; Sigma-Aldrich, St. Louis, MO, USA), and after adding RPMI with 1% penicillin–streptomycin/10% fetal calf serum, the cells were then filtered through a 40-μm cell strainer. Red blood cells were lysed (PharmLyse, BD Biosciences). Different subsets of cell suspension in PBS/5% FCS/2 mM EDTA (2.10^6^ cells/mL) were incubated with Fc block purified rat anti-mouse CD16/CD32 (2.4G2, BD Biosciences) and then with viability dye (Live/Dead, eBioscence) before incubation with a combination of fluorochrome-conjugated anti-mouse antibodies against cell surface markers. The cells were then washed, and when needed, the cells were permeabilized using Cytofix/Cytoperm (BD Biosciences) for intracellular staining. The cells washed with PermWash (BD Biosciences) were then fixed with FACS Lysing 1X (BD Biosciences). Fluorescence-minus-one (FMO) controls were set up for all of the antibodies used. Data were collected using a Fortessa x20 flow cytometer (BD Biosciences) and analyzed with FlowJo software (FlowJo7.6.5 Tree Star, Ashland, OR, USA). The antibodies used for staining are listed in **Supplementary Table S1**.

### Peritoneal, liver, and spleen macrophages

Peritoneal cells were recovered by flushing the peritoneal cavity with 5 mL of ice-cold PBS. Cells (10^5^) were immobilized on slides by Cytospin*™* centrifuge (Sigma 2–7 Cyto). Kupffer cells and splenic macrophages were isolated based on their adhesion properties. Single-cell suspensions from the liver were prepared using the same protocol as for FACS. Splenic macrophages were isolated by directly filtering the spleen through a cell strainer. After washing, splenic and liver isolated cells were plated in RPMI medium supplemented with streptomycin/penicillin and 10% FBS and incubated for 2 h at 37 °C. Cell debris and non-adherent cells were removed by gently washing with PBS.

### Bone-marrow-derived macrophages

Single-cell suspensions from bone marrow were obtained from femurs by PBS flushing and filtration. Bone-marrow-derived macrophages (BMDMs) were obtained after culture for 6 days in Dulbecco’s modified Eagle’s medium (DMEM) supplemented with 2 mM L-glutamine, 25 mM HEPES, 20% heat-inactivated horse serum, 30% L929 cell supernatant containing macrophage-stimulating factor (M-CSF), and streptomycin/penicillin. Macrophages were then harvested in cold PBS and plated (3 × 10^5^ cells/cm^2^ on six-well plates or 2.10^5^ cells on glass slides) in DMEM supplemented with 2 mM L-glutamine, 25 mM HEPES, streptomycin/penicillin, and 2% FBS for overnight incubation. Then, bone-marrow-derived macrophages were stimulated with LPS 100 ng/mL, and at 1, 2, 6, and 24h after the addition of LPS, BMDM cells were fixed in 3% paraformaldehyde solution and then washed with PBS1x. Alternatively, the BMDMS cells were treated with ferric ammonium citrate (100 µM) for 16 h. Non-treated cells were used as controls. The immunofluorescence protocol was the same as described for tissue sections.

### Histology

Tissue samples were fixed with 4% paraformaldehyde, embedded in paraffin, and cut into 5-µm-thick sections, followed by hematoxylin/eosin (H&E) or Perls staining (3.7% hydrochloric acid and 5% potassium ferrocyanide/nuclear Fast Red counterstain) for microscopic analysis. For assessment of ferric iron deposits, Perls staining was further enhanced with 0.5 mM H_2_O_2_ in PBS (pH7.4) for 1 h followed with 3,3′-diaminobenzidine (DAB, Sigma-Aldrich) solution (0.025% DAB/0.12% H_2_O_2_ in PBS (pH7.4)) when indicated. Histological images were acquired with a NanoZoomer NDP scan 1.0.9 scanner and imported into NDP view 2.0 software (Hamamatsu Photonics K.K., Japan).

### Immunofluorescence, TUNEL assay, and ROS

Tissue sections were deparaffinized, and BMDMs were plated on glass slides fixed with 4% paraformaldehyde for 10min. For immunofluorescence, the sections were treated with 50 mM glycine/50 mM NH_4_Cl for 15min at room temperature and blocked with 10% horse serum/2% bovine serum albumin/1 mM CaCl_2_/1 mM MgCl_2_ in PBS (pH7.4)/0.05% Tween 20. The sections were then incubated overnight at 4 °C with the primary antibody (listed in **Supplementary Table S1**). Then, the sections were incubated for 2h at room temperature with Alexa Fluor 488-conjugated and/or Alexa-Fluor 594-conjugated anti-IgG (Thermo Fisher Scientific, Waltham, MA, USA) secondary antibodies. Controls were carried out by the omission of the primary antibodies. Sections were treated with TrueBlack Lipofuscin Autofluorescence Quencher (Biotium, Fremont, CA, USA). Nuclei were stained with 4,6-diamidino-2-phenylindole (DAPI, Invitrogen, Thermo Fisher Scientific), and slides were mounted in anti-fading medium (Fluoromount^®^, Thermo Fisher Scientific).

For the TUNEL assay, terminal deoxynucleotidyl transferase-mediated dUTP nick-end labeling (TUNEL) assay was performed by using Apoptosis Detection Kit (CF5^R^594 tunel assay apoptosis detection kit, Biotium) by which CF^R^594 dye-dUTP incorporation at the free end of the fragmented DNA is visualized by fluorescence microscopy. TUNEL stain was performed according to the manufacturer’s protocol.

For detection of reactive oxygen species (ROS), dihydroethidium (DHE, Sigma-Aldrich), oxidative red fluorescent dye, was used for cytosolic superoxide anion (O_2_^−^) detection in section by oxidation. Upon reaction between ROS and DHE, a red fluorescence was produced, namely, 2-hydroxyethidium. Briefly, deparaffinized sections were incubated in DHE solution (liver: 1 mM, 5min; spleen: 10 μM, 1min) in PBS, following washing with PBS and mild fixation using 1% PFA, for 10min. The stained sections were washed with PBS and mounted with an antifade reagent.

Fluorescent microscopy images were acquired by using a Zeiss Observer Z7 inverted microscope coupled with a Zeiss LSM 980 Airy Scan 2 device and imported into Zeiss ZEN blue 3.1 software (Carl Zeiss Co. Ltd., Jena, Germany). When indicated, immunofluorescence images were acquired by using a Leica CTR6000 confocal microscope (Leica, Heidelberg, Germany) and imported into MetaMorph software (Molecular Devices, Downingtown, PA, USA). For fluorescence analyses, images were acquired under identical imaging settings to ensure reliable quantification. Positive cells on images and mean fluorescence intensities (MFI) were quantified using ImageJ and Zen software from at least three different regions of interest of each sample.

### Cellular labile iron and ROS

The intracellular labile iron pool (LIP) was measured based on a calcein quenching assay in which the labile iron has the ability to bind and quench the fluorescence of the cell-permeable chelator calcein acetoxymethyl ester (CA-AM) in a stochiometric manner. For the iron calcein quenching and ROS assays, cells were plated (10^5^) in 96-well black flat-bottom plates and treated as indicated; after washing, the cells were incubated with 1 µM nonfluorescent calcein-AM for 15min (calcein quenching assay) or 5 mM H_2_DCFDA for 30min (ROS assay) at 37 °C and were then washed before measurement using 480 nm/530 nm and 490 nm/520 nm filter pairs, respectively, in a Fluoroskan Ascent microplate fluorometer (Thermo Fisher Scientific).

### ELISAs

Serum or culture medium of stimulated BMDMs were used to measure cytokines by ELISA according to the manufacturer’s protocol (R&D Systems, Wiesbaden Nordenstadt, Germany).

### Western blot

For western blotting, proteins from snap-frozen tissues or BMDMs were homogenized in a tissue protein extraction reagent (Thermo Fisher Scientific) with protease inhibitor cocktail (Roche, Bâle, Switzerland) by using Precellys homogenizer (Bertin Technologies, Montigny-le-Bretonneux, France). The protein concentration was measured in a NanoDrop spectrophotometer (Thermo Fisher Scientific) at 280 nm. The samples were separated by SDS/PAGE and then electroblotted onto 0.2-µm nitrocellulose membranes (Protran, Amersham Biosciences, Amersham, UK). The membranes were blocked in TBS/0.2%Tween-20/10% skim milk and then incubated sequentially with primary antibodies, followed with horseradish peroxidase-conjugated secondary antibodies. The proteins were detected using a chemiluminescent reagent (either ECL Prime Western Blotting Detection Reagent, GE Healthcare, Little Chalfont, Buckinghamshire, UK or SuperSignal™ West Femto Maximum Sensitivity Substrate, Thermo Fisher Scientific), digitally imaged using iBright 1500 Invitrogen), and quantified using ImageJ software. The antibodies used are listed in **Supplementary Table S1**.

### Total RNA sequencing, data processing, and statistical analysis

RNA was isolated and purified using Qiagen RNeasy Mini Kit. Nanodrop was employed to determine the total RNA concentration, and an aliquot of total RNA was run on a denaturing agarose gel stained with ethidium bromide to assess the integrity. For library construction, we mixed 350 ng of total RNA from three samples, followed by reverse transcription into first-strand cDNA. RNA-cDNA hybrids were quantified using Qubit™ dsDNA HS Assay kit (Invitrogen). Sequencing libraries were prepared using the Oxford Nanopore Technologies (Oxford Nanopore Technologies, Oxford, UK) Direct RNA Sequencing Kit (SQK-RNA004) protocol. Adapters were ligated to the RNA–cDNA hybrids (1 µg). RNA sequencing was performed on the ONT MinION Mk1b sequencer (Oxford Nanopore Technologies), with MinION RNA flow cell (R9.4 version, Oxford Nanopore Technologies), run by MinKNOW version (1.10.16). MinKNOW performs data acquisition, real-time analysis, and basecalling, generates fastsq files, and checks quality control. The EPI2ME Transcriptomics workflow, bioinformatics resources from Oxford Nanopore Technologies, was used to demultiplex, to trim/filter RNA (pychopper v2.7.10), and to map the resultant high-quality reads (minimap2) to the transcriptome sequence reference of ENSEMBL Mus-musculus.GRCm39.cdna.all.fa and Mus-musculus.GRCm39.dna-rm.toplevel.fa; for gene annotation, Mus-musculus.GRCm39.113.gtf was used. Gene-level differential expression analysis was performed using the Bioconductor R (v4.4.3) package DESeq2 (v1.22.2) to generate log_2_(fold change).

We explored Gene Ontology Enrichment by employing R packages analysis. Next, we conducted additional analyses, including the construction of gene expression profile heatmaps based on the exploration of inflammatory-specific and/or stress pathway enrichment. In these analysis approaches, we deliberately employed a gene list intricately linked to inflammation and/or stress. This gene list was meticulously curated by extracting genes associated with the biological process (BP) category of ontology gene sets through keywords from GO. Gene expression levels were quantified as counts, and normalization was carried out using the DESeq2 package using Wald test statistics.

### Quantitative real-time reverse transcription polymerase chain reaction

Total RNA from tissue samples or cells was isolated by TRIzol (Invitrogen). Reverse transcription of 1 µg of total RNA was performed with a SuperScript III First-Strand Synthesis System (Life Technologies) in a total volume of 20 μL. The mRNA levels of *Hamp1* (sequences of the primers, see **Supplementary Table S2**) were measured using a QuantiTect SYBR Green PCR system (Qiagen, Hilden, Germany) and an Aria Mx real-time PCR system (Agilent Technologies, Santa Clara, CA, USA). *Gapdh* quantitect primers from Qiagen were used. qRT-PCR was performed in triplicate, and data analysis was performed using the 2^−ΔΔCT^ method for relative quantification and normalized to the levels of glyceraldehyde 3-phosphate dehydrogenase (GAPDH).

### Statistical analysis

Data were analyzed by using Mann–Whitney test for two groups or one-way ANOVA for several groups followed by Tukey’s post-test with GraphPad Prism 6 (San Diego, CA, USA). In all cases, the results were presented as mean ± standard deviation (SD) and were considered significant, with *p*<0.05.

## Results

### Loss of autophagy in myeloid cells exacerbates LPS-induced shock

To investigate the role of autophagy in myeloid cells during acute inflammation, mice with autophagy deficiency in monocytes/macrophages and granulocytes (*Atg5^f/f^LysM-cre^+^*) and C57BL/6J wild-type mice were subjected to a single intraperitoneal injection (i.p.) of LPS. First, *Atg5^f/f^LysM-cre^+^* and wild-type C57BL/6J mice (*n*=5 per group) were injected with a single dose of 0.5 mg LPS/kg body weight, a dose below the LD_100_ in wild-type C57BL/6J mice, which is known to trigger low-grade inflammation, or injected with vehicle alone. The animals were monitored for 24h after the injection. The *Atg5^f/f^LysM-cre^+^* mice challenged with LPS showed signs of distress (apathy, hunched posture, and ruffled fur) and became moribund within 1 to 24h after LPS injection, with some succumbing to death (three out of five animals; **Supplementary Figure S2A**). In contrast, none of the wild-type mice showed signs of distress or died within 24 h after LPS injection.

**Figure 1:**
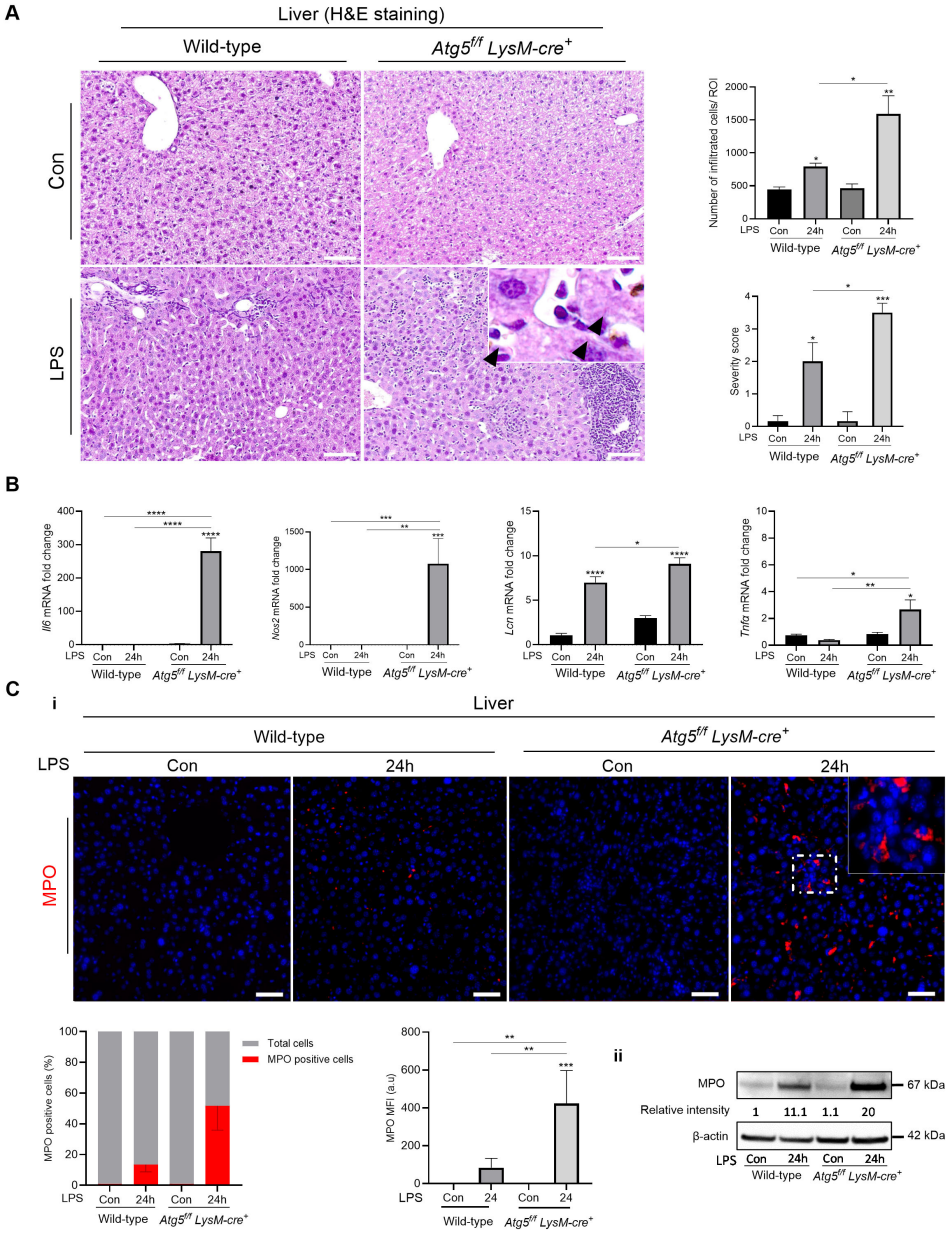
LPS exacerbates inflammation-induced shock in *Atg5^f/f^LysM-cre^+^* mice. **(A)** Liver histology and semi-quantitative analysis of the severity of liver inflammation from *Atg5^f/f^LysM-cre^+^* and wild-type mice after LPS stimulation. Representative liver sections stained with hematoxylin/eosin (H&E); peri-portal and sinusoidal cell infiltration and severity score for inflammation (the scoring table is given in **Supplementary Table S3**) presented as mean per five to six ROIs. *Atg5^fl/fl^ LysM-cre^+^* and wild-type mice received a single intraperitoneal dose of LPS (0.5 mg LPS/kg body weight) or vehicle as control (Con) and were analyzed after 24h. NanoZoomer scan imaging, original magnification ×40; scale bars, 20 µm. The top-right inset in the *Atg5^fl/fl^ LysM-cre^+^* panel shows an enlarged phagocyte with brown hemosiderin deposits (arrow heads) from the same area. **(B)** Liver *Il-6*, *Nos2*, *Lcn2*, and *TNF-α*; mRNA level evaluation performed by qPCR in triplicate for each group (relative to *Gapdh*) expressed as 2^-ΔΔCt^ values in *Atg5^f/f^LysM-cre^+^* and wild-type mice 24h after LPS injection (0.5 mg/kg). **(C)** Detection of MPO in the liver. *Atg5^f/f^LysM-cre^+^* and wild-type mice were analyzed 24h after injection of LPS at 0.5 mg/kg body weight. i: Representative immunofluorescence staining of MPO; composites from MPO (red) and DAPI-stained nuclei (blue) from three animals per group. Zeiss Observer Z7 fluorescence microcopy imaging; original magnification ×20; scale bars, 50 µm. Histograms show the quantification of positive cells counted per region of interest from three mice expressed as percent and mean fluorescence intensity (MFI) of MPO per cell. ii: Liver MPO protein expression and relative intensity to control wild-type mice. Immunoblotting of MPO and β-actin as the loading controls. Representative images from three experiments are shown. Densitometric analysis was performed on the immunoblot shown as well as on immunoblots with lysates from two additional *Atg5^f/f^LysM-cre^+^* and WT mice; after normalization to β-actin in each lane, *Atg5^f/f^LysM-cre^+^* results were normalized to the wild-type control. The data are presented as means ± standard errors. Con, vehicle controls. *p<0.05; **p<0.01; ***p<0.001; ****p<10^-4^.

Histopathological changes were observed in H&E-stained liver sections, the primary target of intraperitoneal LPS challenge, including dilation of the sinusoids and marked infiltration of immune cells, which were more severe in *Atg5^f/f^LysM-cre^+^* mice 24 h after the LPS administration (LPS 0.5 mg/kg, **Figure 1A**). Additionally, the LPS-challenged *Atg5^f/f^LysM-cre^+^* mice exhibited enlarged phagocytes and brown hemosiderin deposits. While some hemosiderin granules were found in the liver parenchyma of both control and LPS-treated mice, extracellular granules were focally distributed around blood vessels specifically in *Atg5^f/f^LysM-cre^+^* mice (**Figure 1A**). Pro-inflammatory mediators *Il-6*, *Lcn2*, and *Nos2* secreted by hepatocytes and known to play central roles in various stages of inflammation were significantly upregulated in *Atg5^f/f^LysM-cre^+^* mice compared to wild-type mice. In contrast, *Tnf-α*, a pro-inflammatory cytokine mainly produced in the liver by Kupffer cells, was only moderately expressed in *Atg5^f/f^LysM-cre^+^* mice (**Figure 1B**). We also noted that these inflammatory mediators were not significantly expressed in the spleen (**Supplementary Figure S2B**).

We also noted that in LPS-treated mice, the red blood cell count was reduced to 8.45 × 10^6^ ± 0.39/mm^3^ in wild-type mice, compared to 10.29 × 10^6^ ± 0.29/mm^3^ in untreated controls (*p* > 0.05). In *Atg5^f/f^LysM-cre^+^* mice, the red blood cell count decreased to 7 × 10^6^ ± 1.15/mm^3^ compared to 9.94 × 10^6^ ± 0.5/mm^3^ in untreated controls (*p* > 10^-3^). The difference between the two genotypes was not statistically significant. The hemoglobin levels were 13.5 ± 1.57 g/dL in LPS-treated wild-type mice (vs. 15.53 ± 0.6 g/dL in untreated controls; not significant); in LPS-treated *Atg5^f/f^LysM-cre^+^* mice, the hemoglobin levels further decreased to 11 ± 1.53 g/dL (*p* > 10–^3^ vs. 14.42 ± 1.04 g/dL in untreated controls; *p* > 0.05 vs. LPS-treated wild-type mice).

Neutrophil infiltration in the liver was further evaluated by assessing myeloperoxidase (MPO) expression. Significantly more MPO*-*positive cells were detected in the liver parenchyma of *Atg5^f/f^LysM-cre^+^* mice, along with higher fluorescence intensity, compared to wild-type controls (**Figure 1C—i).** Moreover, MPO protein levels were significantly elevated in *Atg5^f/f^LysM-cre^+^* mice 24 h after LPS injection (0.5 mg/kg; *p*<0.001; **Figure 1C—ii**). Therefore, the absence of autophagy in macrophages/granulocytes in *Atg5^f/f^LysM-cre^+^* mice exacerbates LPS-induced shock, likely due to an exaggerated systemic inflammatory response.

### Autophagy deficiency in myeloid cells promotes iron dysregulation and ferroptosis in the liver during endotoxemia

Since the LPS dose of 0.5 mg/kg significantly affected the survival of *Atg5^fl/fl^ LysM-cre^+^* mice, the mice were then injected with a single, lower dose of LPS (0.1 mg/kg i.p.), and to dynamically observe changes, the mice were sacrificed 1, 4, and 6 h after injection (*n*=6–10 per group from two independent experiments). At 6 h after LPS injection, both *Atg5^f/f^LysM-cre^+^* and wild-type groups exhibited reduced body weight compared with their respective vehicle-treated control groups (**Supplementary Figure S3A**). In addition, most *Atg5^f/f^LysM-cre^+^* mice exhibited apparent signs of illness (listlessness, hunched posture, puffed-up fur) within 6 h after LPS injection, whereas these symptoms were absent in wild-type mice (**Supplementary Figure S3B**). These findings indicate that autophagy is needed early to mount a proper response to LPS. Functional autophagy in tissues was demonstrated by the expression of Atg5, p62, and Lc3b I/II. Autophagy activation in the liver was confirmed by increased Lc3b-II levels and decreased p62 levels at 4–6 h after LPS stimulation in wild-type mice. In contrast, *Atg5^f/f^LysM-cre^+^* mice showed higher p62 and lower Lc3b-II levels at 6h (**Supplementary Figures S3C**, **S4**). In the spleen, a strong increase in p62 levels was observed from 1h after stimulation in *Atg5^f/f^LysM-cre^+^* mice, whereas the levels remained low in wild-type mice (**Supplementary Figures S3C**, **S4**).

**Figure 2:**
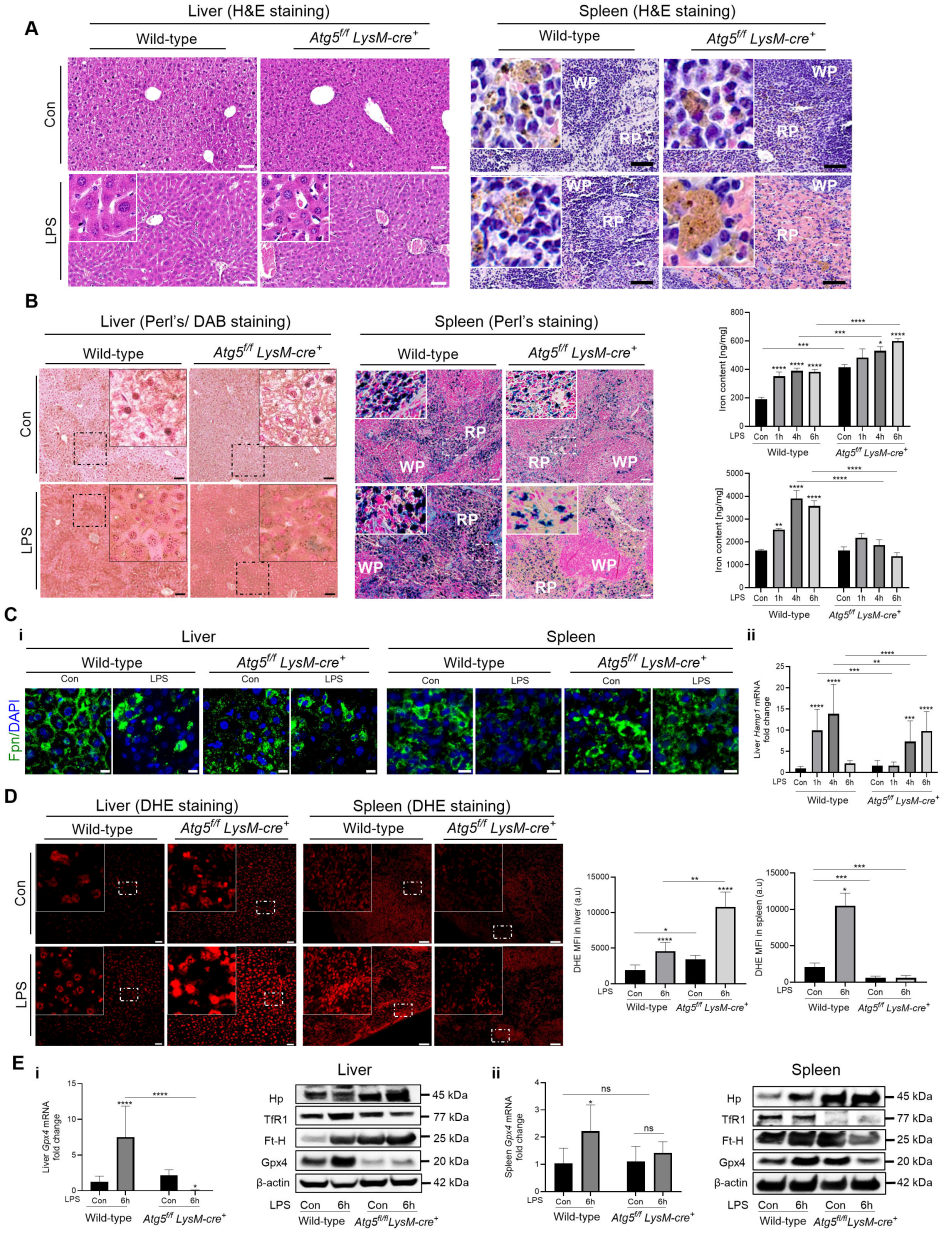
Intraperitoneal LPS challenge results in acute distress in *Atg5^f/f^LysM-cre^+^* mice. *Atg5^f/f^LysM-cre^+^* and wild-type mice were injected intraperitoneally with a single dose of LPS at 0.1 mg/kg body weight. **(A)** Liver and spleen H&E histology after LPS injection. Representative liver and spleen sections from mice 6h after LPS stimulation stained with hematoxylin/eosin (H&E). NanoZoomer scan imaging; original magnification ×40; scale bars, 30 µm. Arrow head, hemosiderin pigments, which appear as small brown punctate staining The inset in the liver panel shows sinusoid red blood infiltration. **(B)** Liver and spleen iron content after LPS injection. Histological examination of non-heme iron and tissue iron content in the liver and spleen of *Atg5^f/f^LysM-cre^+^* and wild-type mice 6h after LPS injection. Non-heme iron deposition was visualized by Perl’s staining (blue) for spleen sections and after DAB enhancement (brown) for liver sections. Representative, with enlarged inset, images are shown from *n*=6–10 per group from two independent experiments. NanoZoomer scan imaging; original magnification ×40; scale bars, 20 µm. Histograms of tissue iron content in the liver and spleen in *Atg5^f/f^LysM-cre^+^* and wild-type mice, as determined by a ferrozine assay and reported as nanogram of iron per milligram of dried tissue. The data are presented as the mean and standard error values; *n*=6–10 per group. WP, white pulp; RP, red pulp. **(C)** i: Detection of ferroportin by immunofluorescence in liver and spleen and hepcidin expression in liver. Detection of ferroportin by immunofluorescence in liver and spleen. Representative images are composites from ferroportin-stained (green) and DAPI-stained (blue) nuclei from four animals per group. For liver, Leica fluorescence microcopy imaging; for spleen, Zeiss Observer Z7 fluorescence microcopy imaging; original magnification ×40; scale bars, 10 µm. ii: Liver hepcidin (*Hamp1)* mRNA levels (relative to *Gapdh*) expressed as 2^-ΔΔCt^ values. **(D)** Assessment of reactive oxygen species (ROS) levels in liver and spleen 6 h after LPS injection. Representative fluorescence microscopic images of intracellular ROS production by DHE peroxide indicator staining (red) in the liver and spleen from three animals per group. Zeiss Observer Z7 fluorescence microcopy imaging, original magnification ×20; scale bars for liver 10 µm and for spleen 15 µm. The histograms below are quantifications of DHE signal; the data present the mean intensity of fluorescence per cell measured in randomly selected fields (ROI) and averaged. Data are expressed as means ± SD of three independent experiments. **(E)** Liver (I) and spleen (ii) *Gpx4* mRNA levels (relative to *Gapdh*) expressed as 2^-ΔΔCt^ values in *Atg5^f/f^LysM-cre^+^* and wild-type mice 6**h** after LPS injection. Liver (I) and spleen (ii) immunoblot of Gpx4, TfR1, Ft-H, Hp, and β-actin as the loading controls. Representative images from three experiments are shown. Relative protein levels were quantified by densitometry in **Supplementary Figure S4**. Con, vehicle controls. *p<0.05; **p<0.01; ***p<0.001; ****p<10^-4^.

In the liver, as early as 6 h after LPS injection, red blood cell infiltration in the sinusoids and venous vessels and hepatic sinusoid congestion were observed (**Figure 2A**, **Supplementary Figure S3D**). The increased hepatocellular injury in *Atg5^f/f^LysM-cre^+^* mice was manifested by higher serum levels of aspartate transaminase (AST, **Supplementary Figure S3E**) and alanine transaminase (ALT, not shown) compared to wild-type mice. Furthermore, the pro-inflammatory mediators (*Il-6*, *Nos2*, *Lcn2*, *Tnf-α*) produced by the liver were more highly expressed in *Atg5^f/f^LysM-cre^+^* mice than in wild-type mice (**Supplementary Figure S3F**). The serum levels of acute-phase proinflammatory cytokines, Il-1β and Il-6, were also elevated in LPS-stimulated *Atg5^f/f^LysM-cre^+^* mice after 6h (**Supplementary Figure S3G**).

The spleen is the organ with the largest population of macrophages. Macrophages in the red pulp are specialized in phagocytosing aging red blood cells and recycling iron, whereas macrophages lining the marginal sinus (marginal zone macrophages (MZMs) and marginal metallophilic macrophages (MMMs)) are involved in immune system activation. The splenic index was significantly higher in untreated *Atg5^f/f^LysM-cre^+^* mice compared to wild-type mice; then, as early as 4 h after LPS injection, the spleen weight tended to increase in wild-type mice (**Supplementary Figure S3H**). The H&E histology showed lighter staining of the splenic macrophages in *Atg5^f/f^LysM-cre^+^* mice and reduced Perls-stainable non-heme iron compared to wild-type controls (**Figures 2A, B**), as previously described (27). As early as 6 h after LPS administration (0.1 mg/kg i.p.), H&E staining revealed severe congestion and swollen splenic macrophages with loss of cellularity and highly translucent cytoplasm. These features were more pronounced in the spleens of *Atg5^f/f^LysM-cre^+^* mice compared to wild-type mice to the extent that the normally clear marginal zones surrounding the white pulp follicles were no longer visible (**Figure 2A**, **Supplementary Figure S3I**). Interestingly, the pro-inflammatory *Il-6*, *Nos2*, *Lcn2*, and *Tnf-α* mediators expressed by the spleen were at lower levels in LPS-treated *Atg5^f/f^LysM-cre^+^* mice compared to wild-type mice (**Supplementary Figure S3J**).

Non-heme iron staining indicated iron accumulation in hepatocytes proximal to the portal area but sparse staining in Kupffer cells in unstimulated *Atg5^f/f^LysM-cre^+^* compared to wild-type mice, as previously reported (27, **Figure 2B**). In both groups of LPS-stimulated mice, iron stored in the liver significantly increased compared to the respective untreated controls, correlating with total liver iron content (**Figure 2B**). Furthermore, a marked increase in Perls staining of non-heme iron in spleen sections and total splenic iron content was observed in LPS-treated wild-type mice compared with untreated control mice. In contrast, in LPS-treated *Atg5^f/f^LysM-cre^+^* mice, Perls-stainable non-heme iron was absent in a large number of splenic macrophages (**Figure 2B**) correlating with low total splenic iron content (**Figure 2B**), thus indicating iron depletion in macrophages. However, large extracellular iron deposits were observed.

In *Atg5^f/f^LysM-cre^+^* mice, although immunodetection of the iron exporter ferroportin in the liver was low, its level in splenic macrophages was higher compared to wild-type mice, as previously reported (27). This observation in *LysM*-*Atg5* ^-/-^ mice correlates with elevated iron release from macrophages and resulting iron accumulation in hepatocytes. In LSPS-treated wild-type mice, consistent with elevated iron levels in both the liver and splenic macrophages, weak immunodetection of the ferroportin at the plasma membrane and punctate intracellular localization in the liver and macrophages of the spleen were observed (**Figure 2C—i**). This result correlated with the increased hepatic expression of hepcidin in LPS-treated animals compared to untreated controls, which likely promoted ferroportin degradation and contributed to the increase in liver iron content (**Figure 2C—ii**). In contrast, LPS-treated *Atg5^f/f^LysM-cre^+^* mice showed weak ferroportin immunodetection at the plasma membrane in the liver and splenic macrophages despite exhibiting low hepatic hepcidin expression (**Figure 2C—i**). Interestingly, splenic macrophages in LPS-treated *Atg5^f/f^LysM-cre^+^* mice also displayed an abnormal membrane morphology (**Figure 2C—i**). Overall, these findings suggested that iron redistribution and elevated iron release from macrophages in *Atg5^f/f^LysM-cre^+^* mice occurred independently of ferroportin regulation and resulted in iron accumulation in hepatocytes.

ROS production correlated with iron levels. Elevated ROS levels were prominently detected in the liver parenchyma of *Atg5^f/f^LysM-cre^+^* mice 6h after LPS treatment. In contrast, LPS-treated wild-type mice exhibited significantly lower hepatic ROS levels compared to *Atg5^f/f^LysM-cre^+^* mice (**Figure 2D**). Conversely, splenic macrophages from LPS-treated *Atg5^f/f^LysM-cre^+^* mice showed significantly reduced ROS levels relative to those from LPS-treated wild-type animals (**Figure 2D**). Glutathione peroxidase 4 (Gpx4), a membrane-associated enzyme, directly eliminates lipid peroxides and acts as a key suppressor of oxidative stress-induced ferroptotic cell death. In wild-type mice, hepatic *Gpx4* mRNA and protein levels significantly increased between 4 and 6 h after LPS treatment (**Figure 2Ei**, **Supplementary Figure S4**). In contrast, no such upregulation was observed in *Atg5^f/f^LysM-cre^+^* mice. Moreover, in *Atg5^f/f^LysM-cre^+^* mice, hepatic TfR1 expression was reduced, while the Ft-H levels were elevated, correlating with increased hepatic iron accumulation. In the spleen, TfR1 expression was nearly absent, and the Ft-H levels were lower in *Atg5^f/f^LysM-cre^+^* mice compared to wild-type mice, consistent with the loss of macrophage integrity and reduced splenic iron content (**Figure 2E**, **Supplementary Figure S4**). These findings suggest that iron accumulation in the liver, likely due to decreased ferroportin-mediated export, combined with impaired antioxidant defense and sustained oxidative stress, may lead to ferroptotic cell death in the livers of *Atg5^f/f^LysM-cre^+^* mice following LPS challenge.

To validate these results, BMDMs were isolated from control naïve mice and treated *in vitro* with LPS (100 ng/mL, 1 to 24h). The results confirmed that LPS is a potent inducer of autophagy in macrophages, as evidenced by the induction of Atg5 expression and Lc3b lipidation in BMDMs from wild-type mice exposed to LPS (**Supplementary Figure S5A**). In control BMDMs, Lc3b was not immunodetected; however, in wild-type BMDMs, LPS exposure triggered the formation of numerous Lc3b-enriched vesicles positive for ferroportin, indicating the degradation of ferroportin (**Supplementary Figure S5B**). The cytosolic pool of chelatable iron, i.e., the labile iron pool (LIP), was measured by the quenching of calcein fluorescence, and the ROS levels were evaluated in BMDMs. LPS treatment induced an increase in labile iron level and ROS in BMDMs from wild-type mice, while these levels were lower in cells from *Atg5^f/f^LysM-cre^+^* mice (**Supplementary Figure S5C**). Therefore, by inducing autophagy, LPS triggered the degradation of ferroportin and iron accumulation, which was associated with increased ROS levels. The Atg5^-/-^ cells exhibited lower labile iron and lower ROS levels compared to wild-type BMDMs treated with LPS.

### Macrophage deficient in autophagy induces macrophages’ cell death

**Figure 3:**
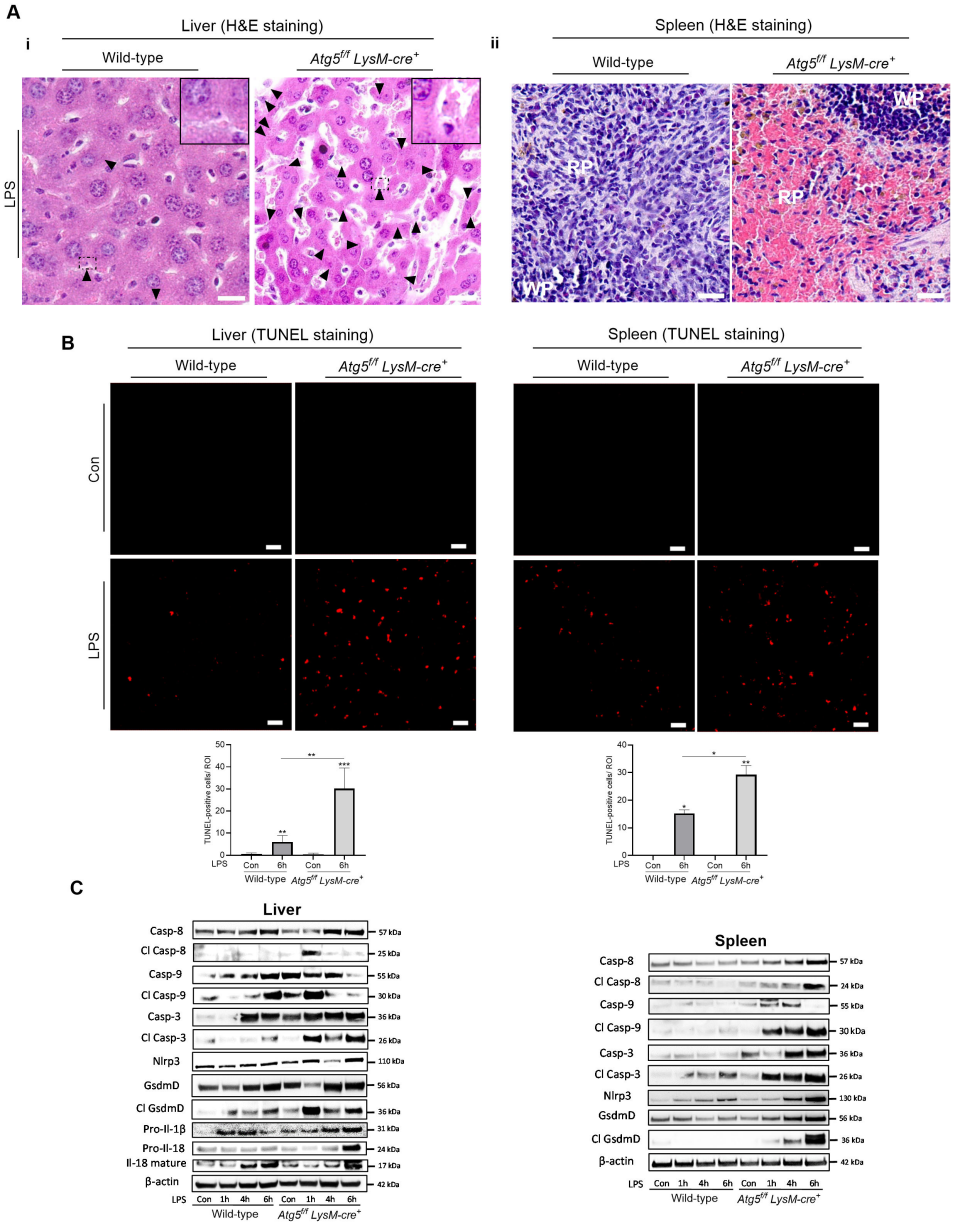
Autophagy-deficient macrophages increases cell death. *Atg5^f/f^LysM-cre^+^* and wild-type mice were injected intraperitoneally with a single dose of LPS at 0.1 mg/kg body weight. **(A)** Liver apoptotic cells and sublytic/lytic splenic macrophages in *Atg5^f/f^LysM-cre^+^* mice after LPS treatment. Liver (I) and spleen (ii) sections stained with hematoxylin/eosin (H&E) analyzed 6h after the injection of LPS. The arrowhead in liver sections indicate small and rounded apoptotic hepatocyte nuclear condensation and fragmented nuclei. NanoZoomer scan imaging, original magnification ×40x; scale bars, 10 µm for liver and 20 µm for spleen. The histological staining panels are representative from *n*=6–10 per group from two independent experiments. **(B)** Apoptotic cells identified by nuclear fluorescence TUNEL staining. Representative TUNEL-stained image for the detection of apoptotic cells and histograms of the number of TUNEL-positive cells per region of interest (ROI) in the liver (I) and spleen (ii) 6h after the injection of LPS. Representative images from three to four animals per group. Zeiss Observer Z7 fluorescence microcopy imaging, original magnification ×20; scale bars, 20 µm. **(C)** Immunoblots of total protein from liver (I) and spleen (ii) from *Atg5^f/f^LysM-cre^+^* and wild-type, untreated control (Con) analyzed 1, 4, and 6 h after the injection of LPS. Western blots were probed for proform and cleaved caspase-8 (Casp-8), caspase-9 (Casp-9), caspase-3 Casp-3), gasderminD (GdsmD), and Il-18 as well as Nlrp3 and Il-1β. The images of the cleaved proteins are longer exposures of the same immunoblots as for pro-forms. To confirm equal loading of the samples, the blots were reprobed with the β-actin antibody. Relative protein levels were quantified by densitometry in **Supplementary Figure S4**. Con, vehicle controls. *p<0.05; **p<0.01; ***p<0.001.

Liver sections from LPS-treated mice (0.1 mg/kg, 6h) revealed an increase in apoptotic features in hepatocytes, such as condensed and fragmented nuclei, and apoptotic bodies, compared to tissues from unstimulated mice (**Figure 3A—i**). The TUNEL assay confirmed a significant increase in the number of TUNEL-positive nuclei in the liver of LPS-treated *Atg5^f/f^LysM-cre^+^* mice compared to the control mice, whereas only a few TUNEL-positive nuclei were observed in the liver of wild-type treated mice (**Figure 3B—i**). The macrophages of the spleen (and from the liver) from LPS-treated *Atg5^f/f^LysM-cre^+^* mice exhibited increased cell volume and a highly translucent cytoplasm, suggesting altered plasma membrane integrity and the release of cellular contents, features indicative of lytic cells (**Figure 3A—ii**). The TUNNEL assay showed few number of positive cells in both groups of LPS-treated mice (**Figure 3B—ii**).

To investigate the cell-death-inducing effects of LPS in mice deficient for autophagy in macrophages, the expression of several markers of regulated cell death—including caspase-3, caspase-8, caspase-9, and gasdermin D and the proinflammatory cytokines Il-1β and Il-18—was examined. In the liver of LPS-treated wild-type mice, the initiator pro- and cleaved-caspase 9 increased at 6 h. Meanwhile, the cleaved caspase-3 apoptotic effector showed a slight increase, and the proinflammatory and pyroptotic effector gasdermin D, as well as the cleaved Il-18, significantly increased between 4 and 6 h (**Figure 3Ci**, **Supplementary Figure S2**). In LPS-treated *Atg5^f/f^LysM-cre^+^* mice, both initiator-cleaved caspase-9 and caspase-8 increased at 1 h, along with a significant rise in cleaved caspase-3, effector of apoptosis, and high levels of cleaved gasdermin D and Il-18 were observed (**Figure 3Ci**, **Supplementary Figure S4**). Overall, the results indicated a stronger induction of apoptosis, in agreement with the TUNEL assay results, as well as pyroptosis pathways in the liver of *Atg5^f/f^LysM-cre^+^* mice compared to wild-type mice.

In the spleen of LPS-treated wild type mice, an increase in cleaved caspase-3 was observed (4–6 h). In contrast, in the spleen of *Atg5^f/f^LysM-cre^+^* mice, high levels of Nlrp3 and cleaved forms of caspase-8, caspase-9, and gasdermin D were detected (**Figure 3Cii**, **Supplementary Figure S4**). The proteolytic maturation of gasdermin D likely forms functional pores in the plasma membrane, promoting the release of cytokines (Il-18 and Il-1β) and facilitating iron fluxes. Furthermore, the increase in gasdermin D activation correlated with morphological changes in macrophages. Specifically, we observed iron depletion in macrophages in both the liver and the spleen, which are typically iron-loaded. Based on these findings, we hypothesize that labile iron was released through gasdermin-D-mediated pores. These results were confirmed in LPS-treated BMDM. The expression levels of caspase-3 (at 4, 6, and 24 h), caspase-9 (at 1h), and cleaved Il-18 (at 24h) proteins were higher in Atg5-deficient macrophages than in wild-type cells (**Supplementary Figures S4**, **S5D**).

### Deficient autophagy in macrophages promotes a proinflammatory macrophage polarization

First, we compared the dynamics of leukocytes in peripheral blood between *Atg5^f/f^LysM-cre^+^* and wild-type mice after treatment (LPS 0.1 mg/kg). A marked reduction in leukocyte counts was observed as early as 1 h after LPS injection (**Supplementary Figure S6**). At 4 h after LPS injection, the wild-type mice exhibited increased the percentages of circulating eosinophils, monocytes, and neutrophils, along with a decrease in the number of lymphocytes, compared to untreated controls. Furthermore, there were higher percentages of circulating neutrophils in the peripheral blood of *Atg5^f/f^LysM-cre^+^* mice (37.7 ± 8%) at 4h after LPS injection compared to wild-type mice (21 ± 5%) (**Supplementary Figure S6**).

**Figure 4:**
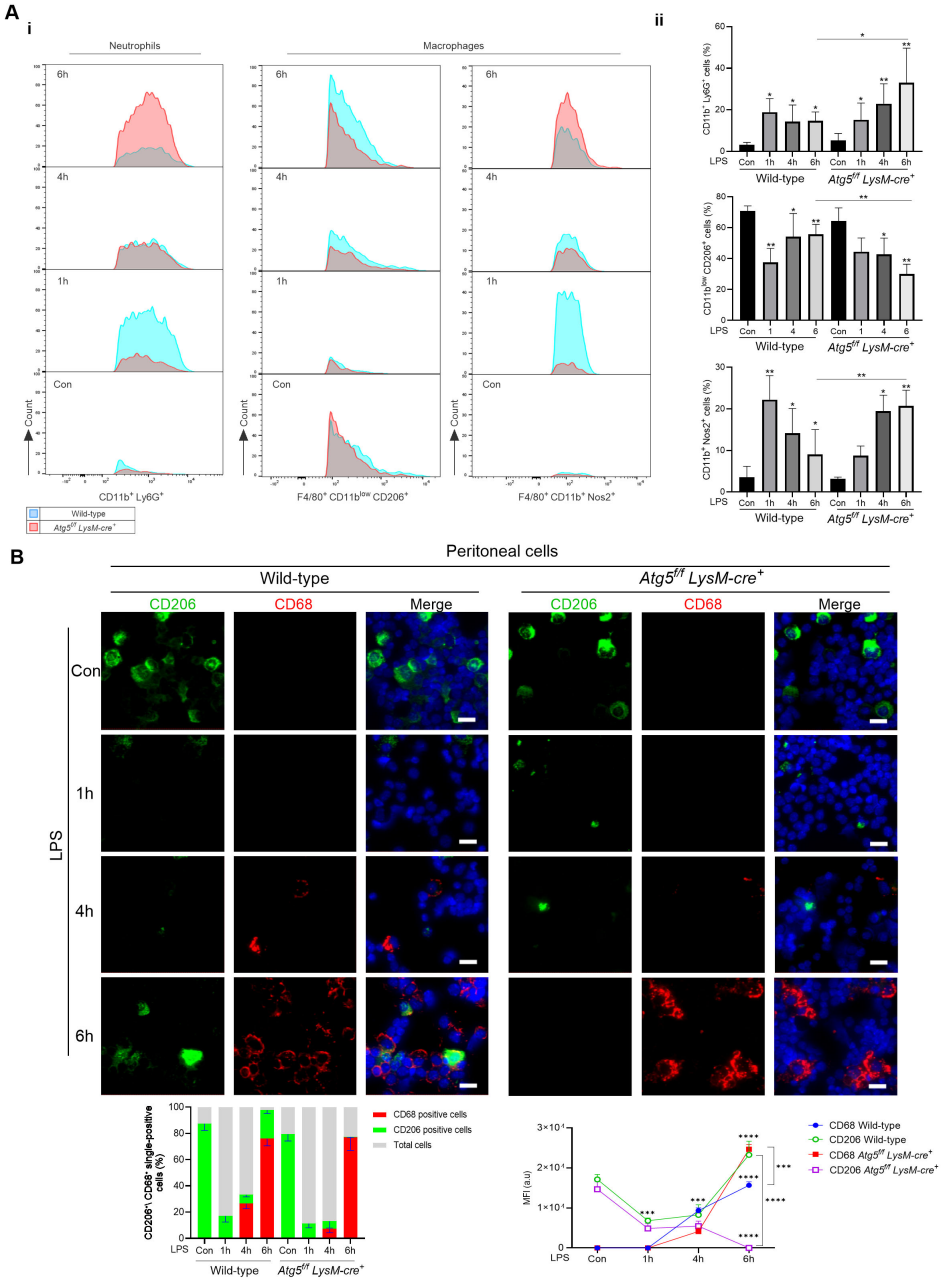
*Atg5^f/f^LysM-cre^+^* mice influences immune cell infiltration in the liver and macrophage polarization following LPS administration. **(A)** Phenotypic analysis of neutrophils and macrophages by flow cytometry. Expression of markers in mice treated with LPS (0.1 mg/kg) and vehicle as controls, analyzed by flow cytometry in liver tissue homogenates. i: Representative overlap histograms showing CD45^+^ Siglec-F^-^ CD11b^+^ Ly6G^+^ neutrophils, CD45^+^ Siglec-F^-^ F4/80^+^ either CD11b^low^ CD206^+^ or CD11b^+^ Nos2^+^ macrophages; blue, wild-type mice; red, *Atg5^f/f^LysM-cre^+^* mice. ii: A quantitative comparison is shown as bar graphs representing the percentage of parent population, i.e., CD45^+^ for neutrophils and CD45^+^ F4/80^+^ for macrophages, respectively. The data shown were obtained from two independent experiments, each including four to six mice per group, and are presented as mean ± SEM. For the analysis of myeloid cells, the surface staining includes fluorochorome-conjugated anti-mouse antibodies for the detection of CD45, F4/80, Ly6G, Siglec-F, CD11b, CD206, and intracellular Nos2. The cells were gated as described in the representative plots of **Supplementary Figure S7**, and the antibodies used are listed in **Supplementary Table S1**. **(B)** Representative images of immunofluorescence staining of peritoneal cells for CD68 (red), CD206 (green), and nuclei stained with DAPI (blue). Zeiss Observer Z7 fluorescence microcopy imaging, original magnification ×20; scale bars, 20 μm. The histograms below were quantifications of positive cells counted using Zen in randomly selected fields and averaged. *p<0.05; **p<0.01; ***p<0.001; ****p<10^-4^.

Next, the total number of mononuclear cells was quantified in the liver by FACS during LPS stimulation (0.1 mg/kg). Along with enhanced egress of immune cells from the periphery, LPS promoted the infiltration of circulating immune cells into the liver and activation of resident macrophages. The CD45^+^ SiglecF^-^ CD11b^+^ Ly6G^+^ neutrophils markedly increased 1 h after LPS injection (43%) and then decreased by approximately 17% after 6 h in wild-type mice (**Figure 4A**). In contrast, in *Atg5^f/f^LysM-cre^+^* mice, the number of infiltrated neutrophils increased progressively to reach 82% at 6 h after LPS injection, consistent with myeloperoxidase (MPO) expression observed in mice 24 h after 0.5 mg/kg LPS injection.

In control mice, the resident macrophage population CD45^+^ F4/80^+^ CD11b^low^ was CD206^-^positive. After LPS stimulation, the CD45^+^ F4/80^+^ population included CD11b^low^ cells and CD11b^+^ subpopulations. The CD45^+^ F4/80^+^ cells were further classified based on their expression of CD206 and Nos2, corresponding to M1-activated macrophage marker signature. In wild-type mice, at 1h after LPS injection, the CD45^+^ F4/80^+^ population increased to 10.8%, while the CD11b^+^ Nos2^+^ (M1) increased from 2.8% to 22.5% of the CD45^+^ F4/80^+^ population, and at 6h after LPS injection, the CD45^+^ F4/80^+^ decreased to 10.8% and CD45^+^ F4/80^+^ CD11b^+^ Nos2^+^ cells represented 18.2 ± 6.5% of the CD45^+^ F4/80^+^ population (**Figure 4A**). Consistent with these findings, the immunofluorescence analysis of peritoneal cells revealed high staining with CD206 antibody before LPS injection, followed by a decrease in CD206 staining and a concomitant increase in staining with the CD68 phagocytic M1 marker at 4h (**Figure 4B**). CD68-positive staining persisted at 6 h after LPS injection, but few cells were CD206-positive.

In *Atg5^fl/fl^ LysM-cre^+^*-stimulated mice, at 1h after LPS injection, the CD45^+^ F4/80^+^ population increased to 16%, while the CD45^+^ F4/80^+^ CD11b^+^ Nos2^+^ (M1)-activated macrophages increased from 2.7% to 8.1% of the CD45^+^ F4/80^+^ population. While the CD45^+^ F4/80^+^ cells increased by 12% after 6 h, 25% of the CD45^+^ F4/80^+^ population were CD45^+^ F4/80^+^ CD11b^+^ Nos2^+^ M1 macrophages (**Figure 4A**). Immunofluorescence staining analysis of peritoneal cells showed a marked increase of CD68-positive cells at 6 h (**Figure 4B**). Therefore, the CD45^+^ F4/80^+^ CD11b^+^ Nos2^+^ M1 macrophage polarization was stronger in *Atg5^f/f^LysM-cre^+^* stimulated compared to wild-type mice.

To confirm these results, bone-marrow-derived macrophages (BMDMs) were isolated from naïve *Atg5^f/f^LysM-cre^+^* and wild-type mice and stimulated with LPS. Before LPS treatment, BMDMs were positive for CD206. The number of positive cells decreased 1 h after the addition of LPS (**Supplementary Figures S8A—i, ii**). In parallel, BMDMs became Nos2-positive, with the mean fluorescence intensity of Nos2 staining per cell being higher in BMDMs from *Atg5^f/f^LysM-cre^+^* mice. Notably, the level of Nos2 expression remained elevated up to 72 h following LPS addition (**Supplementary Figures S8A—i, ii**). In addition, following LPS stimulation, macrophages deficient in Atg5 showed enhanced Tnf-α and Il-1β secretion into the culture media compared to wild-type macrophages (**Supplementary Figure S8B**). To ascertain the influence of iron on macrophage polarization, BMDMs were treated with ferric ammonium citrate from *Atg5^f/f^LysM-cre^+^* mice treated with ferric ammonium citrate for 16 h and showed a lower mRNA expression of a series of M2 markers (*Retlna*, *Mrc1* and *Il-10*) as well as a higher *Tnfα* expression (**Supplementary Figure S8C**).

### RNA-Seq analysis confirms stress-related changes in mice deficient in autophagy in macrophages

**Figure 5:**
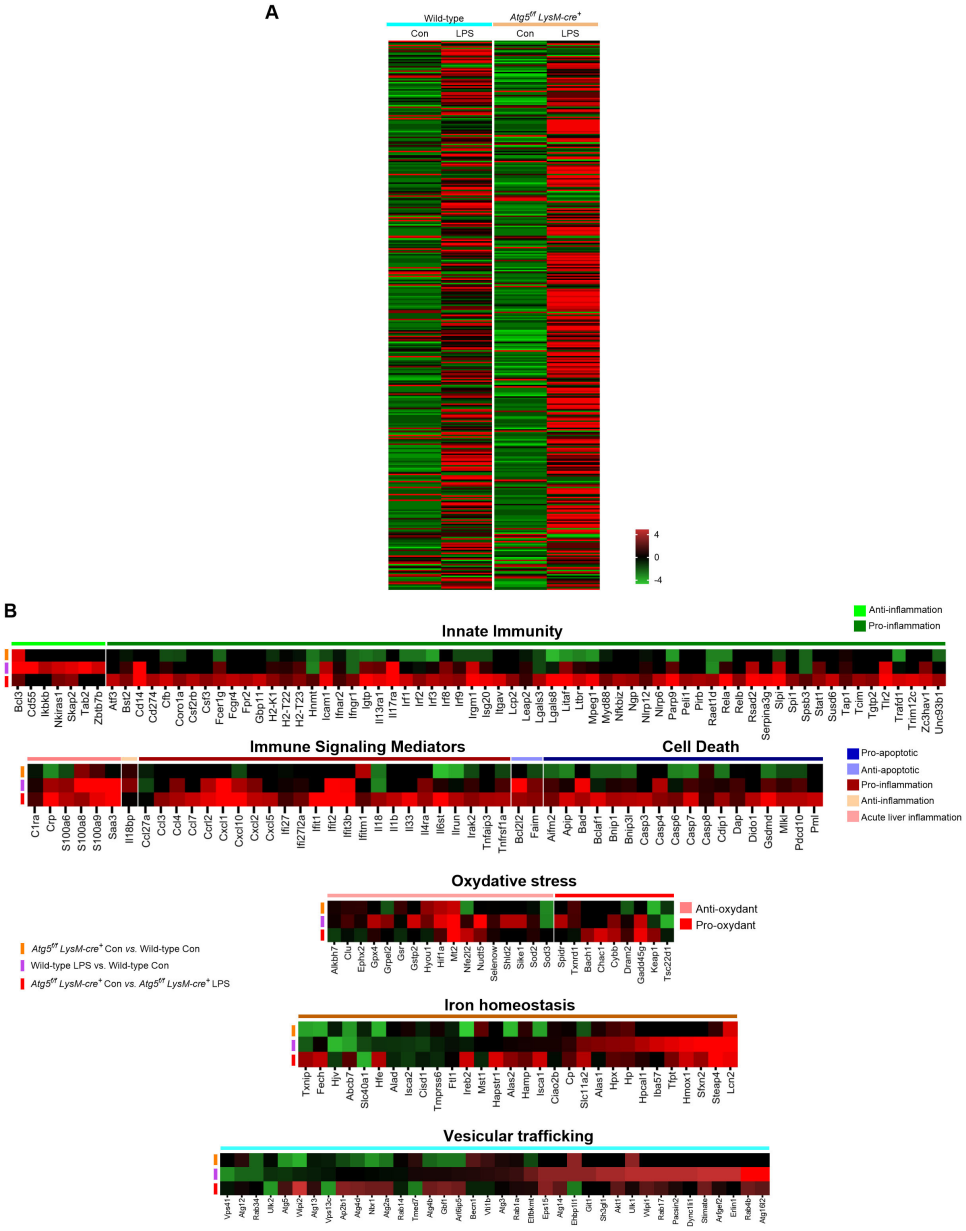
Gene expression profiling by RNA seq analysis and qPCR in stimulated *Atg5^f/f^LysM-cre^+^* and wild-type mice. **(A)** Heatmap of gene expression in LPS-stimulated *Atg5^f/f^LysM-cre^+^* and wild-type mice and unstimulated control groups, as indicated. **(B)** Heatmap of differentially expressed genes comparing the LPS-stimulated *Atg5^f/f^LysM-cre^+^* and wild-type mice to the control mice. Transcriptional profile alterations of the inflammatory and integrated stress response genes selectively arranged were visualized. In this analysis, we performed RNA sequencing on mouse liver from controls and LPS-challenged (6h) mice containing three whole-tissue transcript samples from each group. The screening threshold for the differentially expressed genes (with Wald test statistics) is set to: |log_2_(fold change)| > 1 and *P*-value <0.05. Red indicates upregulation, and green indicates downregulation in either of the LPS-induced models.

To examine the inflammatory response more precisely, we performed RNA sequencing on liver samples from control and LPS-stimulated mice (6 h post-injection). The heatmap, based on standard analyses of our RNA-seq datasets, showed that most of the genes studied exhibited either similar expression levels in both groups or a lower expression in *Atg5^f/f^LysM-cre^+^* mice. Notably, consistent with iron accumulation and increased ROS, several genes related to oxidative stress and/or iron metabolism were differentially regulated between *Atg5^f/f^LysM-cre^+^* and wild-type mice. Specifically, *Hif1a*, haptoglobin (*Hp*), hemopexin (*Hpx*), lipocalin-2 (*Lcn2*), and *Slc11a2*, which encodes the divalent metal transporter 1, were clearly upregulated, whereas *Slc40a1* (encoding ferroportin) and *Keap1* (encoding cytosolic inhibitor of Nrf2) were downregulated in *Atg5^f/f^LysM-cre^+^* mice compared to wild-type mice (**Figure 5A**).

In the LPS-stimulated mice, the heatmap showed a large number of differentially expressed genes. Most of these genes were upregulated (68% and 78% for wild-type and *Atg5^f/f^LysM-cre^+^*, respectively), while only a few were suppressed in LPS-stimulated samples compared to samples from unstimulated mice. To further explore the transcriptional profile associated with LPS-induced inflammatory response in *Atg5^f/f^LysM-cre^+^* mice compared to wild-type mice, we focused on genes assigned to the “inflammation response” and “integrated stress response” gene ontology (GO) categories (394 genes of the 16,587 core gene list).

Several genes were significantly upregulated or downregulated in both *Atg5^f/f^LysM-cre^+^* and wild-type challenged mice (i.e., Wald test statistics, *p*<0.05). Notably, these included a set of transcripts encoding acute-phase proteins, such as C-reactive protein (CRP) and serum amyloid A proteins, as shown in **Figure 5B**. While overlapping upregulation or downregulation patterns were observed, a series of genes assigned to GO “inflammation” and the “integrated stress response” were differentially regulated between *Atg5^f/f^LysM-cre^+^* and wild-type LPS-challenged mice (**Figure 5B**). Key findings included the overexpression of pro-inflammatory pathway (*Rela*, *Myd88*, *Irf1*, and *Nlrp6*) and cytokines (Il1*β*, *Il18*, *Il6st*, *Tnfap3*, *Parp9*, and *Stat1*), as well as chemokines, together with the downregulation of anti-inflammatory genes (*Ikkb*, *Cd55*, *Tab2*) in the liver of LPS-treated *Atg5^fl/fl^ LysM-cre^+^* compared to wild-type mice. The elevated expression of genes associated with the pyroptosis pathway (*Casp3, GsdmD*) and the downregulation of anti-apoptotic gene (*Bcl2l2*) in *Atg5^fl/fl^ LysM-cre^+^* compared to wild-type LPS-stimulated mice corroborated the increase in cell death in the *Atg5^f/f^LysM-cre^+^* mice.

A series of genes from various stimulus-associated pathways were also differentially regulated in LPS-stimulated mice. In particular, genes involved in vesicular trafficking displayed a relatively significant differential expression—for example, members of the *Rab* and *Atg* families, which are involved in phagocytosis, autophagy, and efferocytosis, were significantly upregulated in wild-type LPS-stimulated mice compared to *Atg5^fl/fl^ LysM-cre^+^* mice (**Figure 5B**). Furthermore, genes encoding acute-phase proteins such as haptoglobin (*Hp*) and hemopexin (*Hpx*), lipocalin-2 (*Lcn2*), and heme oxygenase (*Hmox*) were clearly upregulated in LPS-stimulated wild-type mice. In the *Atg5^f/f^LysM-cre^+^* mice, genes related to iron metabolism, including *Lcn2*, *Hamp1*, and *Slc11a2* were upregulated, whereas *Slc40a*, encoding ferroportin cellular iron exporter, was downregulated. This gene expression pattern is consistent with enhanced iron sequestration compared to wild-type mice (**Figure 5B**). Interestingly, the expression of several antioxidant genes, such as *Hif1α* and *Sod2*, was reduced in LPS-treated *Atg5^f/f^LysM-cre^+^* mice despite elevated hepatic iron accumulation and ROS levels (**Figure 5B**). This also includes *Gpx4*, as already mentioned, a key regulator whose downregulation is associated with ferroptotic cell death.

## Discussion

**Figure 6:**
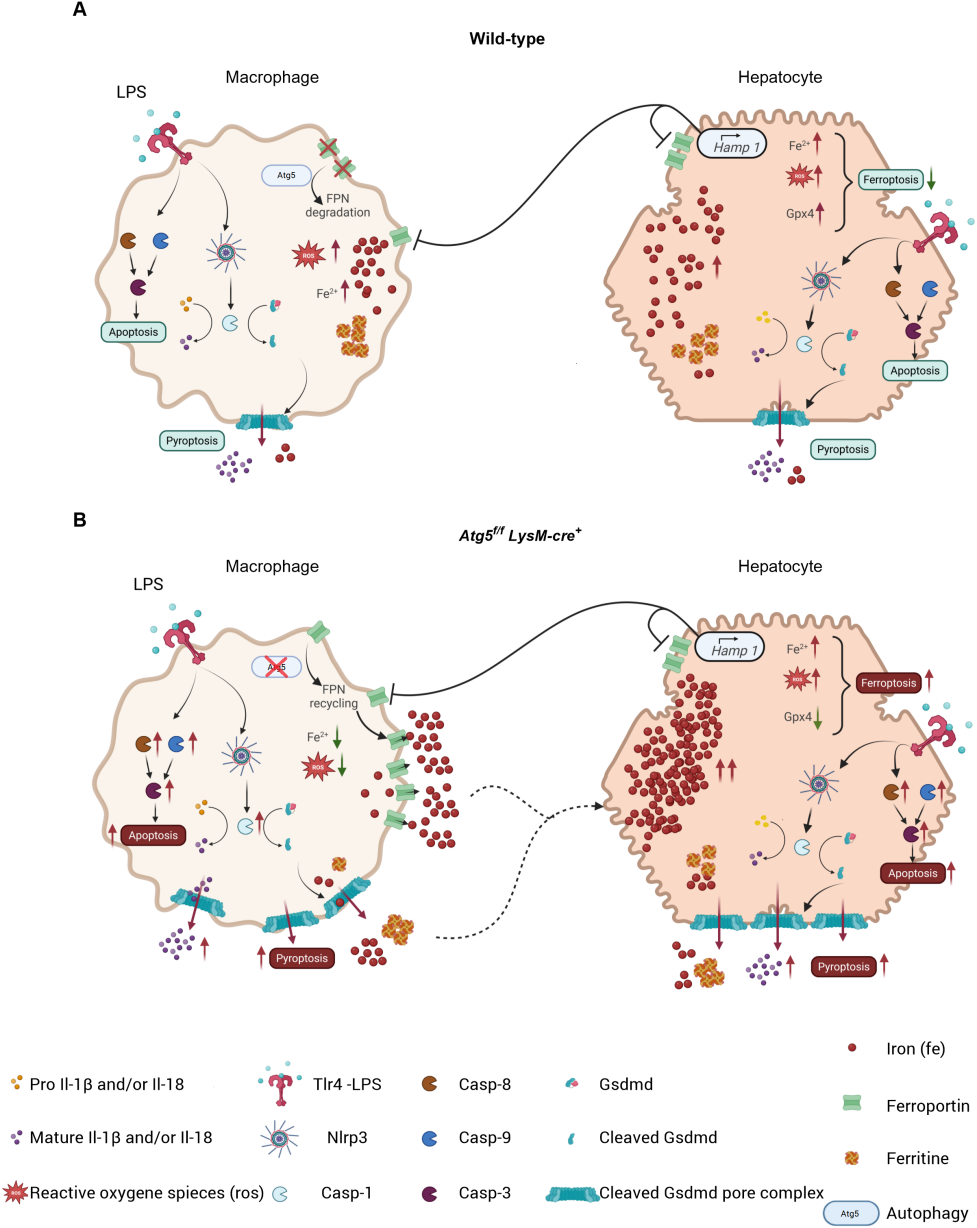
In wild-type mice **(A)**, macrophages exhibit Atg5-dependent autophagy, leading to ferroportin degradation and reduced iron release. This process is accentuated by Hamp1 production in hepatocytes, which promotes ferroportin degradation in both cell types. The resulting increase in intracellular iron promotes the generation of reactive oxygen species (ROS); however, the presence of antioxidant proteins such as Gpx4 prevents ferroptosis by converting ROS into non-toxic molecules. Consequently, apoptosis and pyroptosis are reduced. In *Atg5f/f LysM-cre+* mice **(B)**, macrophages undergo apoptosis and pyroptosis, accompanied by reduced ROS levels due to ferroportin recycling, resulting in increased iron storage in hepatocytes. With increased accumulation of iron and ROS, these hepatocytes show impaired apoptosis, pyroptosis, and ferroptosis resulting from the absence of Gpx4. Symbols represent various proteins and processed involved.

Here we show that a single low dose of LPS is detrimental to mice with myeloid-specific autophagy deficiency compared to wild-type C57BL/6 mice, indicating that macrophage autophagy plays a protective role against severe acute systemic inflammation. LPS-stimulated *Atg5^f/f^LysM-cre^+^* mice exhibited distinct pathological features, including marked vascular congestion, elevated levels of acute-phase proinflammatory cytokines (Il-1β and Il-6), and unique tissue alterations characterized by specific patterns of ROS and iron distribution, compared to wild-type mice. Notably, LPS-stimulated *Atg5^f/f^LysM-cre^+^* mice displayed pronounced iron depletion in splenic macrophages and Kupffer cells, which correlated with increased iron accumulation and ROS levels in hepatocytes. In addition, in the liver of *Atg5^f/f^LysM-cre^+^* mice, ferroptosis and apoptosis cell death were observed, while macrophages in both the spleen and the liver exhibited enhanced pyroptosis (**Figure 6**).

LPS-stimulated mice exhibited iron redistribution and associated ROS production, though this occurs differentially in *Atg5^f/f^LysM-cre^+^* and wild-type mice. While the role of ROS in host tissue damage is well established, its specific contribution to the pathogenesis of SIRS remains insufficiently characterized. Upon inflammation, ROS are primarily generated during the respiratory burst by infiltrating neutrophils and activated macrophages, notably through the production of hydrogen peroxide (H_2_O_2_) and the enzymatic activity of myeloperoxidase (MPO) (28). Additionally, there is a strong interplay between iron and the generation of damaging ROS. The Fenton reaction—mediated by the interaction of H_2_O_2_ with redox active iron or ferri-heme—further amplifies ROS production. As a result, the intracellular labile iron (Fe²^+^) pool is tightly controlled by iron-binding proteins such as ferritin and by the iron exporter ferroportin. Macrophages play a crucial role in regulating systemic iron flux, and in this study, several genes involved in iron metabolism and ROS detoxification were dysregulated in *Atg5^f/f^LysM-cre^+^* mice. These included *haptoglobin*, *hemopexin*, *heme oxygenase*, and iron transporter (*Slc40a1*, *Slc11a2*) expression, which contribute to iron sequestration and clearance of redox-active heme iron (29). In wild-type mice, LPS treatment induced strong iron sequestration in splenic macrophages, accompanied by increased ROS levels and iron deposition in hepatocytes. In contrast, *Atg5^f/f^LysM-cre^+^* mice displayed pronounced iron depletion and reduced ROS production in splenic macrophages, along with increased iron accumulation and elevated ROS in hepatocytes. The balance of intracellular ROS is regulated by antioxidant defense systems (catalase (CAT), superoxide dismutase (SOD), glutathione peroxidase (GPX)). Among these, Gpx4 plays a crucial role in protecting cells from lipid peroxidation, thereby preventing cell death induced by cytoplasmic or mitochondrial ROS. Disruption of Gpx4 function can promote ferroptosis, a form of regulated cell death, typically triggered by the accumulation of toxic lipid peroxides (30). Here, in the liver of *Atg5^f/f^LysM-cre^+^* mice, *Gpx4* expression was significantly reduced, suggesting that it may contribute to ferroptosis cell death.

Moreover, *Atg5^f/f^LysM-cre^+^* mice exhibited a higher number of apoptotic cells following LPS stimulation, accompanied by elevated levels of cleaved caspase-3, compared to wild-type mice. Notably, intracellular ROS have been shown to activate caspase-9 (31, 32), a key decision maker which triggers intrinsic apoptosis. Indeed the increased ROS levels observed in hepatocytes may stem from iron overload and, in turn, could activate caspase-3-mediated apoptosis, a non-lytic and generally anti-inflammatory form of cell death that preserves plasma membrane integrity. Furthermore, caspase-9 has non-apoptotic function; its expression, or exogenous H_2_O_2_, has a positive role in the regulation of autophagy (32–34). Hence, in wild-type mice, autophagy would block the apoptotic pathway.

We previously demonstrated that macrophage autophagy plays a critical role in regulating the turnover of ferroportin at the plasma membrane (27). Loss of autophagy in macrophages results in enhanced iron export, leading to intracellular iron depletion and increased extracellular hemosiderin deposition. In the present study, *Atg5^f/f^LysM-cre^+^* mice stimulated with LPS exhibited iron-depleted macrophages alongside elevated ROS levels and pronounced iron accumulation in hepatocytes. While ROS exert bactericidal effects, they also act as signaling molecules and second messengers, modulating immune responses and contributing to the polarization of immune cells. ROS influence macrophage and neutrophil activation by modulating inflammation-associated signaling pathways (35). Notably, loss of macrophage autophagy enhanced inflammatory cell infiltration in the liver and promoted polarization toward a pro-inflammatory M1 phenotype. Flow cytometric analysis revealed that the population of CD45^+^ F4/80^+^ CD11b^+^ NOS2^+^ (M1) macrophages—characterized by strong phagocytic activity, high ROS production, and cytokine secretion (36)—rose sharply in wild-type mice 1 h after LPS challenge but declined by 4–6 h. In contrast, *Atg5^f/f^LysM-cre^+^* mice showed a delayed yet sustained increase in this subset, peaking at 4 h and remaining elevated at 6 h.

The spleen of LPS-stimulated *Atg5^f/f^LysM-cre^+^* mice exhibited an increased expression of caspase-8 and gasdermin D along with characteristic morphological changes such as swollen and translucent splenic macrophages. These features indicate the enhanced activation of pyroptotic cell death pathway in response to LPS challenge. Caspase family proteases are central regulators of programmed cell death not only in apoptosis but also in non-apoptotic forms such as necroptosis, pyroptosis, and autophagy. Caspase-8, an initiator caspase, triggers inflammasome/gasdermin D-mediated pathway, particularly in professional phagocytes. This pathway converges on the activation and release of mature pro-inflammatory cytokines Il-1b and Il-18 and induces pyroptosis (37, 38). Unlike apoptosis, which preserves membrane integrity and is generally considered anti-inflammatory, the gasdermin D-mediated pathway promotes sublytic membrane disruption, triggering pro-inflammatory responses through the extracellular release of Il-1β and Il-18 cytokines. Ultimately, this can lead to cytolytic pyroptosis. In *Atg5^f/f^LysM-cre^+^* mice, LPS stimulation resulted in pronounced gasdermin D activation and elevated Il-1β and Il-18 expression in both the spleen and liver, correlating with enhanced inflammatory response and more extensive hepatic injury. Moreover, LPS-stimulated *Atg5^f/f^LysM-cre^+^* mice displayed iron-depleted splenic macrophages. These observations suggest that gasdermin D pore formation facilitates both cytokine secretion and intracellular iron efflux, particularly in the absence of ferroportin-mediated export, thereby contributing to local iron dysregulation within the tissue microenvironment. While this process plays an essential role in host defense and infection resolution, uncontrolled pyroptosis can be detrimental due to excessive and sustained inflammatory response. Overall, our data suggest that hepatic macrophages in autophagy-deficient mice undergo hyperpolarization toward an M1 phenotype and that macrophage pyroptosis contributes to prolonged and exacerbated inflammatory responses.

Our study supports the hypothesis that autophagy in myeloid cells exerts a protective role during inflammation. The observed disparities between LPS-treated *Atg5^f/f^LysM-cre^+^* and wild-type mice suggest that the absence of autophagy in myeloid cells has various implications. Our findings highlight that autophagy regulates macrophage polarization, influences neutrophil phenotype, and modulates various regulated cell death pathways, thereby shaping the inflammatory response. Macrophages are key players in the initiation, maintenance, and resolution of inflammation by producing a broad spectrum of mediators, including pro-inflammatory factors (e.g., Il-6, Il-12, Il-1β, Il-18, Tnf-α, ROS) and anti-inflammatory factors (e.g., Il-10, antioxidants, protease inhibitors), and through regulated cell death pathways which contribute differently to the inflammatory process (pro-inflammatory pyroptosis and necrosis; and anti-inflammatory apoptosis and autophagy, which support the controlled removal of inflammatory cells). In our study, we observed significant alterations in macrophage phenotypes in both the liver and spleen of *Atg5^f/f^LysM-cre^+^* mice. In the liver, key pro-inflammatory mediators—Nos2, Il-6, Tnf-α, and Lcn2—were significantly upregulated as early as 6 h after LPS administration, with further increases observed at 24 h in *Atg5^f/f^LysM-cre^+^* mice. Indeed activated Kupffer cells in the liver secrete cytokines, particularly Il-6 and Tnf-α, which are primary inducers of acute-phase protein (APP) gene expression. This response was markedly enhanced in *Atg5^f/f^LysM-cre^+^* mice compared to wild-type mice, potentially exacerbating the pro-inflammatory immune response. In contrast, in the spleen—where the large population of red pulp macrophages are involved in clearing senescent red blood cells and recycling iron and marginal zone macrophages contribute to immune activation by bridging innate and adaptive immunity—these pro-inflammatory mediators were not significantly upregulated. However, the alterations in splenic macrophage polarization may have disrupted the balance between pro-inflammatory and anti-inflammatory signals. The altered splenic macrophage phenotype contributed to iron redistribution, reduced hemoglobin levels, and decreased red blood cell count observed in *Atg5^f/f^LysM-cre^+^* mice 24 h after LPS challenge. Additionally, increased ferroptosis in these mice may have further amplified inflammation through the release of pro-inflammatory factors. Moreover, the accumulation of apoptotic macrophages, combined with defective autophagy, may have impaired the efficient clearance of pro-inflammatory immune cells, thereby sustaining the pro-inflammatory environment.

### Limitations of the study

We observed altered iron distribution, accompanied by iron depletion in macrophages of *Atg5^f/f^ LysMcre^+^* mice upon LPS challenge. Since most circulating iron originates from macrophage-mediated recycling, this suggests a functional disruption of autophagy-deficient macrophages. However, although our study primarily focused on macrophage profiling, we do not exclude the potential contribution of autophagy in neutrophils to the severe immune response to LPS.

A panel of markers for the detection of iron distribution, ROS, cell death, and inflammation was assessed by staining tissue sections. Our findings showed statistically significant differences between *Atg5^f/f^ LysMcre^+^* and wild-type mice. We also found that Atg5 deficiency enhances macrophage cell death and promotes a pro-inflammatory state compared to the wild-type genotype. However, the methods used do not allow for simultaneous or co-localized analysis, which limits the interpretation of the results. To investigate whether these processes occur in the same cells, sensitive and integrated approaches, such as flow cytometry-based assays, may provide greater precision.

Our results showed that apoptosis, pyroptosis, and ferroptosis were more strongly induced in *Atg5^f/f^ LysMcre^+^* mice than wild-type mice after LPS treatment. However, we were unable to determine whether these types of cell death occurred in the same cells or in distinct cell populations, nor could we decipher the primarily role of each pathway. The use of specific inhibitors (e.g., caspase inhibitors for apoptosis and pyroptosis, liproxstatin for ferroptosis) would help provide more detailed insights.

## Data Availability

The data presented in the study are deposited in the NCBI repository, accession number PRJNA1344263.
