# Supplementary material for: Disrupted macrophage autophagy as a driver of cell death and LPS-induced lethal shock in systemic inflammation
Source: Front Immunol. 2025 Oct 23;16:1610033. doi: 10.3389/fimmu.2025.1610033 (PMC12589025; doi:10.3389/fimmu.2025.1610033)

Supplemental Figure 1

A, **Atg5 expression**. The protein expression of Atg5 was detected using western blotting in bone marrow derived macrophages (BMDMs), isolated macrophages from spleen and the liver, and total liver extract from wild-type and *Atg5$^{fl/fl}$ LysM-cre^{+}* mice. β-actin was used for loading control. Floxed mice which harbor exon 3 of the *Atg5* gene flanked by two loxP sites and are heterozygous for *lysozyme M cre* did not show expression of Atg5 protein in BMDMs and isolated macrophages, but expressed Atg5 in the liver.

Supplemental figure 1

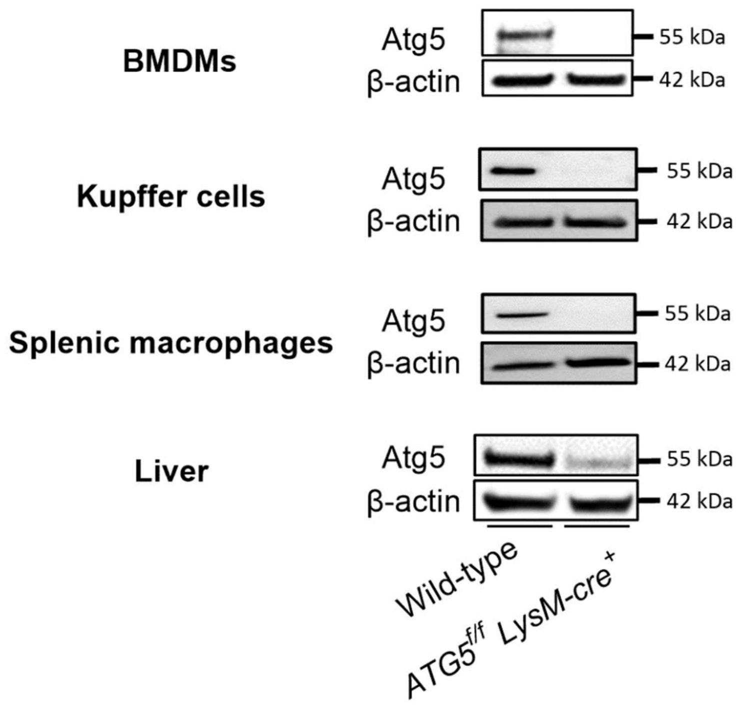

Supplement: Supplementary file 1 [file DataSheet1.pdf]
