# Supplementary material for: Disrupted macrophage autophagy as a driver of cell death and LPS-induced lethal shock in systemic inflammation
Source: Front Immunol. 2025 Oct 23;16:1610033. doi: 10.3389/fimmu.2025.1610033 (PMC12589025; doi:10.3389/fimmu.2025.1610033)

Supplemental Figure 2

Atg5$^{fl/fl}$ LysM-cre^{+} and wild-type mice were injected intraperitoneally with a single dose of LPS at 0.5 mg/kg body weight or vehicle as control (Con), and analyzed at 24 hours after injection. **A,** Survival curve of wild-type and *Atg5$^{f/f}$LysM-cre^{+}* mice treated with either LPS or vehicle. Mice were treated with LPS (0.5 mg/kg body weight) or vehicle by intraperitoneal injection. The mice were divided into four groups: wild-type vehicle group (WT Con, n=5), wild-type LPS group (WT LPS, n=5), *Atg5$^{f/f}$LysM-cre^{+}* vehicle group (Atg5 Con, n=5), and *Atg5$^{f/f}$LysM-cre^{+}* LPS group (Atg5 LPS, n=5). The statistical analysis method we used was Kaplan-Meier log-rank test. **B,** Spleen *Il-6*, *Nos2*, *Lcn2*, and *TNF-α*, mRNA levels evaluation performed by qPCR in triplicate for each group (relative to *Gapdh*) expressed as $2^{-\Delta\Delta Ct}$ values in *Atg5$^{f/f}$LysM-cre^{+}* and wild-type mice 24 h after LPS injection (0.5mg/kg). Con, vehicle controls. $*p<0.05$.

Supplemental figure 2

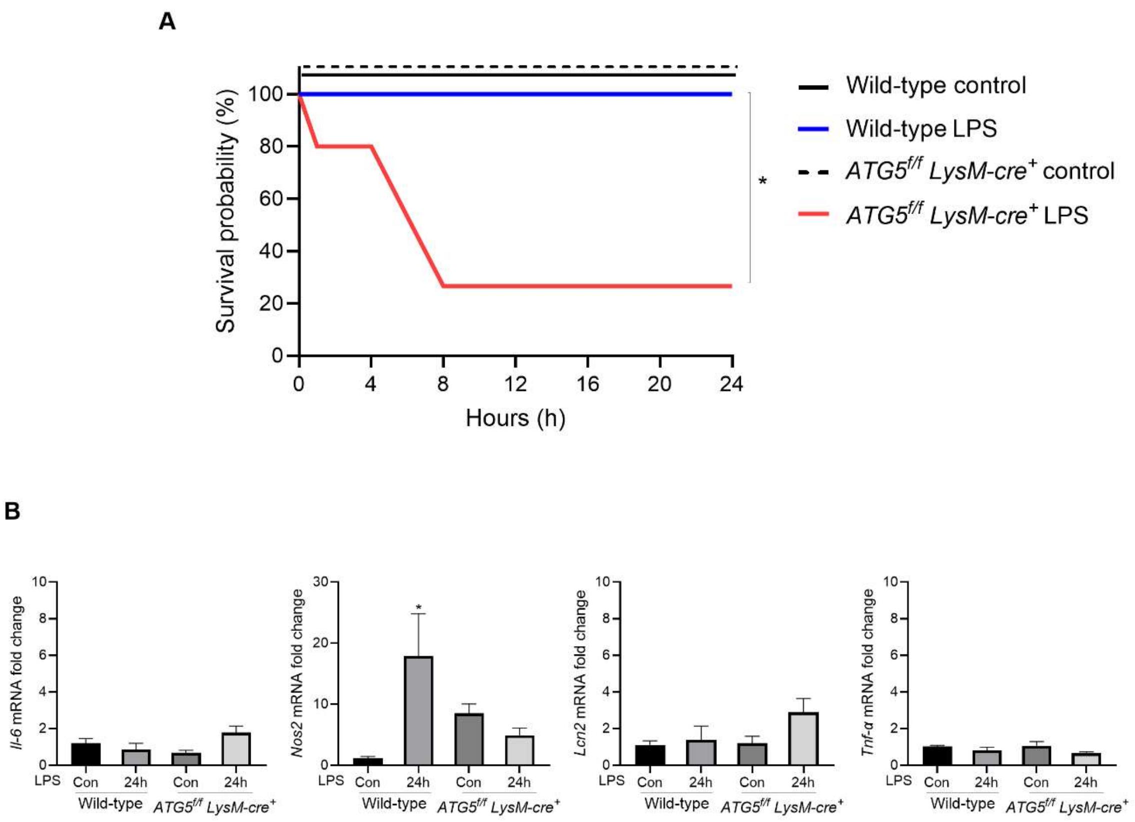

Supplement: Supplementary file 2 [file DataSheet2.pdf]
