# Supplementary material for: Disrupted macrophage autophagy as a driver of cell death and LPS-induced lethal shock in systemic inflammation
Source: Front Immunol. 2025 Oct 23;16:1610033. doi: 10.3389/fimmu.2025.1610033 (PMC12589025; doi:10.3389/fimmu.2025.1610033)

Supplemental Figure 3

Atg5$^{fl/fl}$ LysM-cre^{+} and wild-type mice were injected intraperitoneally with a single dose of LPS at 0.1 mg/kg body weight or vehicle as control (Con), and analyzed at 1, 4 and 6 hours after injection.

A, Body weight after LPS treatment. Histogram of body weight of *Atg5$^{f/f}$LysM-cre^{+}* and wild-type mice presented as the mean, with dots indicating individual measurements; Data (n=4-6 per group) from a representative of two independent experiments are presented as the mean ± S.D.

B, Clinical evaluation after LPS injection. *Atg5$^{f/f}$ LysM-cre^{+}* and wild-type mice. The clinical evaluation included animal appearance, mobility, and fur ruffling, with for each a score for assessing severity from good =0 to worse =2.

C, Autophagy activation. The protein expression of p62, Atg5 and Lc3b I and II were detected using western blotting in the liver and spleen from wild-type and *Atg5$^{fl/fl}$ LysM-cre^{+}* mice. β-actin was used for loading control. Relative protein levels were quantified by densitometry in Supplemental Figure 4).

D, Semi-quantitative analysis of the severity of liver inflammation from *Atg5$^{f/f}$LysM-cre^{+}* and wild-type mice after LPS stimulation. Severity score was quantified from liver sections stained with hematoxylin/eosin (H&E), data are presented as mean per 5-6 ROI (scoring Table is given in Supplemental Table 3).

E, Serum aspartate transaminase (AST). Histogram of the mean and standard error values with dot plots of serum AST levels. Data (n=4-6 per group) from a representative of two independent experiments are presented as the mean ± S.D.

F, Liver *Il-6*, *Nos2*, *Lcn2*, and *TNF-α*, mRNA levels evaluation performed by qPCR in triplicate for each group (relative to *Gapdh*) expressed as $2^{-\Delta\Delta Ct}$ values in *Atg5$^{f/f}$LysM-cre^{+}* and wild-type mice**.**

G, Histogram of serum cytokine levels. ELISA results for inflammatory cytokine Il-6, Il-1β, and Il-10 production in serum. Data (n=4-6 per group) from a representative of two independent experiments are presented as the mean ± S.D.

H, Spleen index. Spleen weight-to-body weight (%) ratios of *Atg5$^{f/f}$ LysM-cre^{+}* and wild-type mice.

I Semi-quantitative analysis of the severity of spleen inflammation from *Atg5*$^{f/f}$*LysM-cre*$^{+}$ and wild-type mice after LPS stimulation. Red pulp cell count and severity score was quantified from spleen sections stained with hematoxylin/eosin (H&E), data are presented as mean per 5-6 ROI (scoring Table is given in Supplemental Table 3).

J, Spleen *Il-6*, *Nos2*, *Lcn2*, and *Tnf-α*, mRNA levels evaluation performed by qPCR in triplicate for each group (relative to *Gapdh*) expressed as $2^{-\Delta\Delta Ct}$ values in *Atg5*$^{f/f}$*LysM-cre*$^{+}$ and wild-type mice**.**

Con, vehicle controls. Data are presented as histograms of the mean and standard error values, with individual measurements; n=4-6 in each group. *p < 0.05, **p=0.01, ***p<0.001, ****p<10^{-4}.

Supplemental figure 3

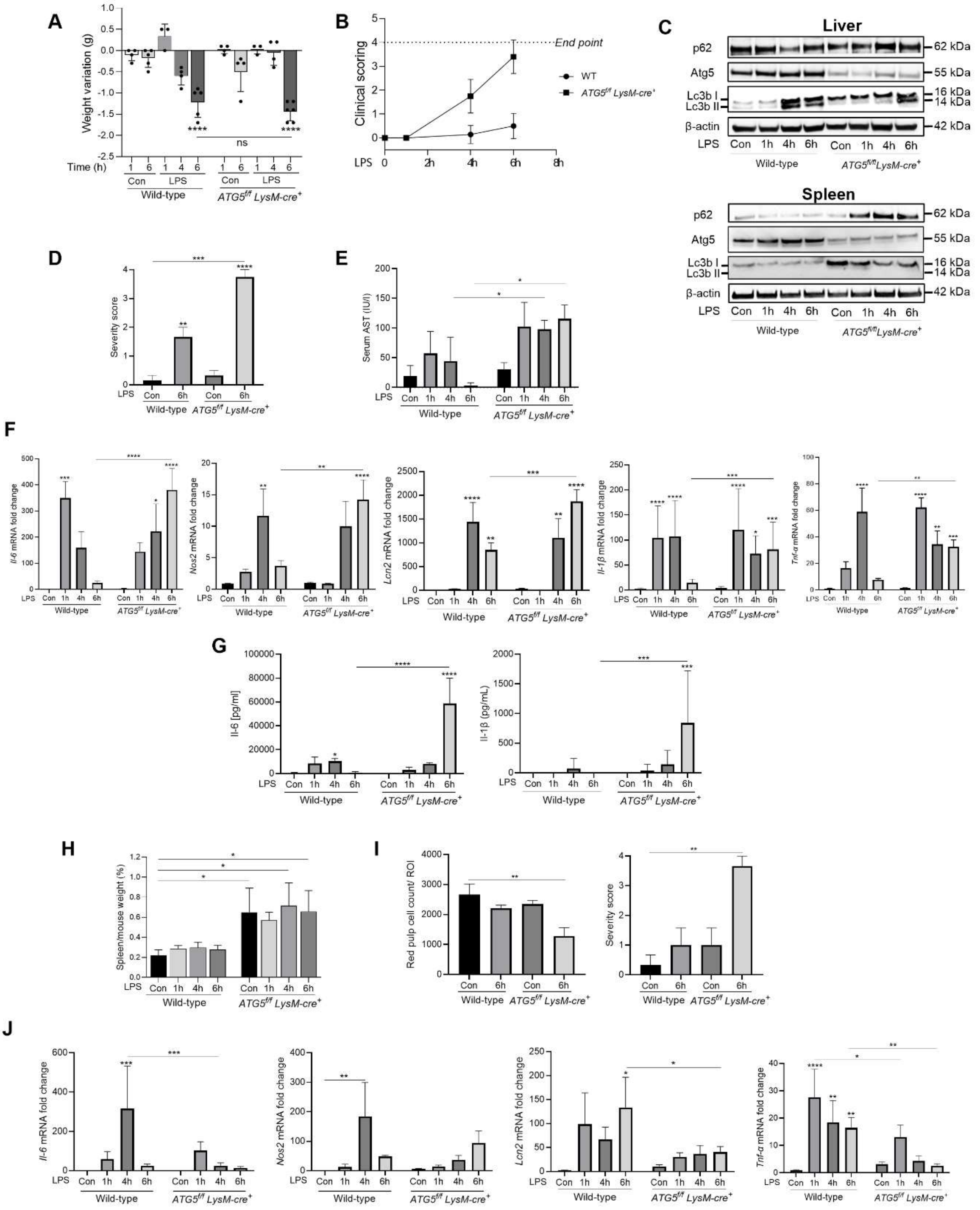

Supplement: Supplementary file 3 [file DataSheet3.pdf]
