# Supplementary material for: Disrupted macrophage autophagy as a driver of cell death and LPS-induced lethal shock in systemic inflammation
Source: Front Immunol. 2025 Oct 23;16:1610033. doi: 10.3389/fimmu.2025.1610033 (PMC12589025; doi:10.3389/fimmu.2025.1610033)

Supplemental Figure 4

Immunoblots of total protein from liver, spleen, and BMDMs from *Atg5$^{f/f}$LysM-cre^{+}* and wild-type, untreated control (Con), and 6 hours after injection of LPS 100 µg/kg. Western blots were probed for proform and cleaved caspase-8 (Casp-8), caspase-9 (Casp-9), caspase-3 Casp-3), gasderminD (GdsmD), and Il-18, as well as p62, Atg5, Lc3b I/II, Nlrp3 Il-1β, Gpx4, TfR1, Ft-H, and Hp. The images of the cleaved proteins are longer exposures of the same immunoblots as for pro-forms. To confirm equal loading of the samples, the blots were reprobed with the β-actin antibody. Relative protein levels quantified by densitometry are indicated below. Densitometric analysis was performed on the immunoblot shown as well as on immunoblots with lysates from 2 additional *Atg5$^{f/f}$LysM-cre^{+}* and wild-type mice; after normalization to β-actin in each lane, *Atg5$^{f/f}$LysM-cre^{+}* results were normalized to the wild-type control. The data are presented as means. Con, saline controls.

Supplemental figure 4

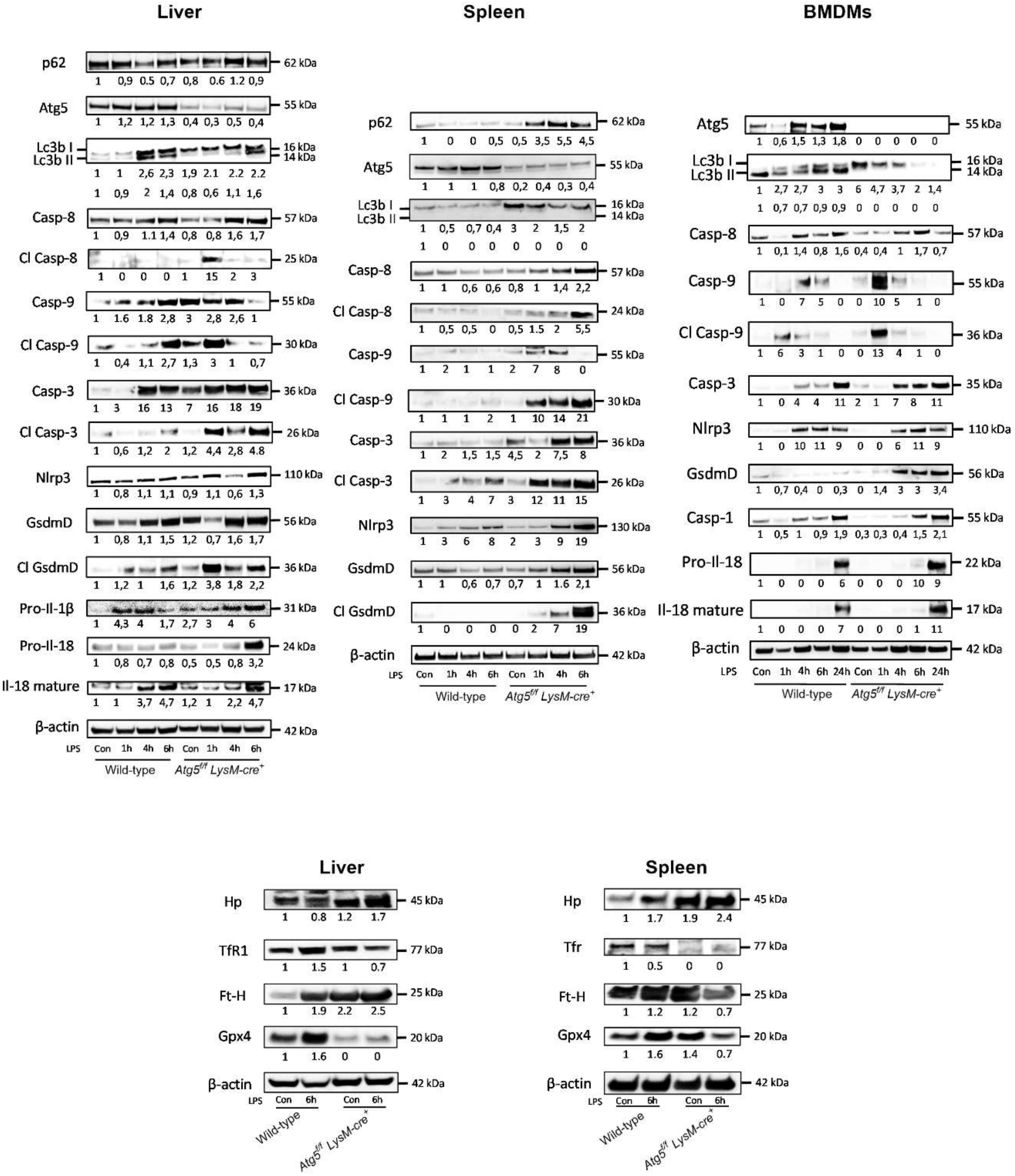

Supplement: Supplementary file 4 [file DataSheet4.pdf]
