# Supplementary material for: Disrupted macrophage autophagy as a driver of cell death and LPS-induced lethal shock in systemic inflammation
Source: Front Immunol. 2025 Oct 23;16:1610033. doi: 10.3389/fimmu.2025.1610033 (PMC12589025; doi:10.3389/fimmu.2025.1610033)

Supplemental Figure 5

BMDMs exposed to LPS (100 ng/mL) for the indicated time.

A, Immunoblots of total protein. BMDMs were exposed to LPS or vehicle (Con) and homogenates of cells were used to examine the Atg5 and Lc3b protein expression levels. Representative western blots for n=2-4 (quantification of protein by densitometry in Suppl Figure 4). **B**, Immunofluorescence colocalization of ferroportin and Lc3b in BMDMs from *Atg5$^{f/f}$LysM-cre^{+}* and wild-type mice treated with LPS. BMDMs were double-labeled with a rabbit anti-Fpn antibody and Alexa Fluor 488-conjugated secondary anti-rabbit IgG (green), followed by rabbit anti-Lc3 antibody and Alexa Fluor 594-conjugated secondary anti-rabbit IgG (red). The cell nuclei were stained with DAPI (blue). Zeiss Observer Z7 fluorescence microcopy imaging; original magnification 40x. The representative images shown are from 2 independent experiments. **C**, Intracellular labile iron pool (LIP) and ROS in BMDMs. Cells ($10^{4/}$ 96 plate well) were seeded and Hoechst 33342 intensity was used to quantify the number of cells after treatment. Calcein quenching assay for labile iron pool (LIP) measurement in BMDMs from *Atg5$^{f/f}$LysM-cre^{+}* and wild-type mice. H_2DCFDA fluorescence assay for measurement of the ROS in BMDMs from *Atg5$^{f/f}$LysM-cre^{+}* and wild-type mice. Representative analyses are shown. For calcein, the mean fluorescence intensity (MFI) of cells incubated with calcein was normalized to Hoechst intensity. ROS production is represented as the percentage relative to Hoechst intensity. **D**, Deficient autophagy increased LPS-induced expression of cell death markers in BMDMs. BMDMs were exposed to LPS and homogenates of cells were used to examine the protein expression levels of Caspase-1, -3, -8, -9, Gsdm D, Nlrp3, and Il-18. Representative western blots (quantification by densitometry in Suppl Figure 3). Con, vehicle controls. ∗p<0.05; ∗∗p<0.01; ∗∗∗p<0.001; ****p<10^{-4}.

Supplemental figure 5

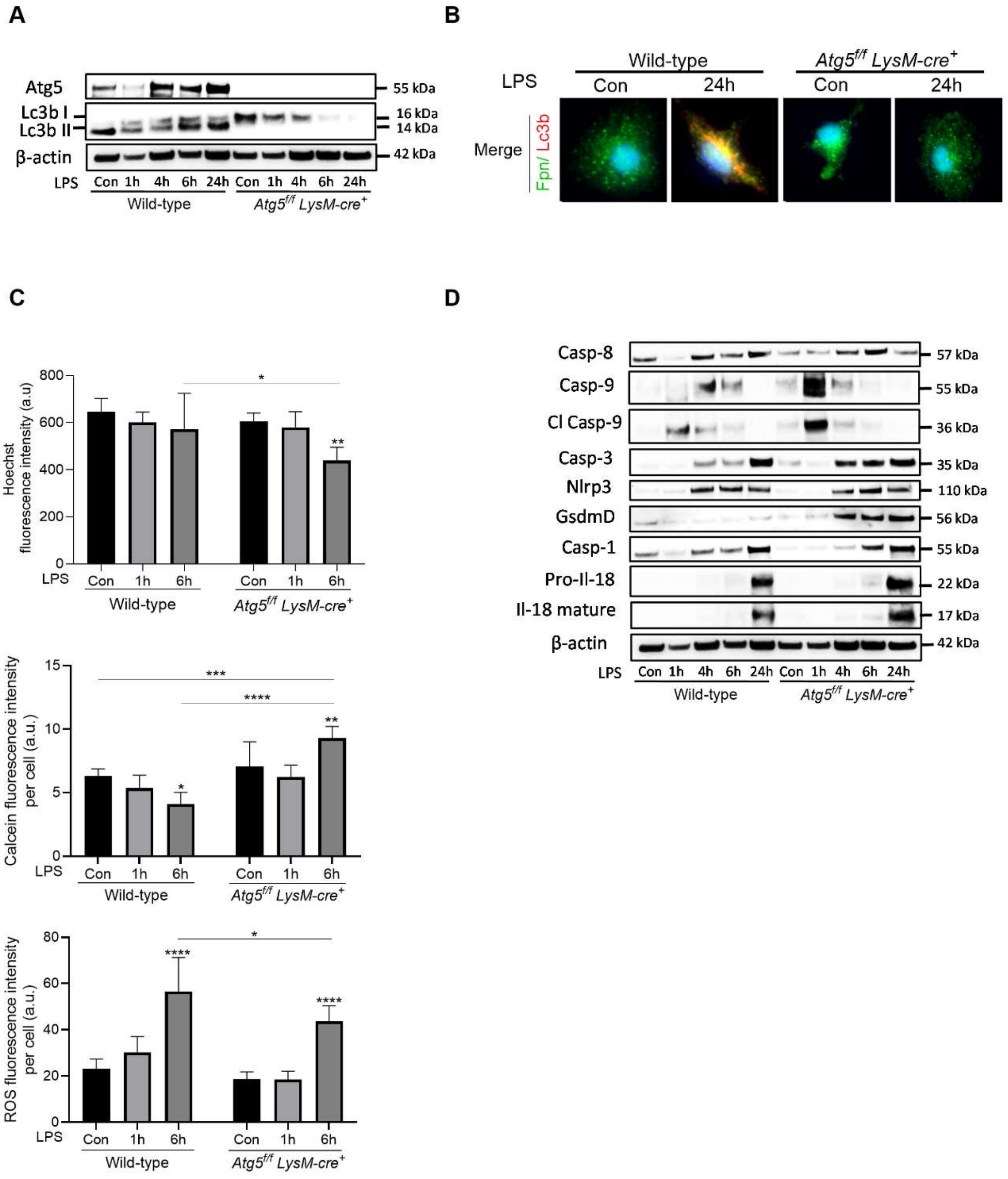

Supplement: Supplementary file 5 [file DataSheet5.pdf]
