# Supplementary material for: Disrupted macrophage autophagy as a driver of cell death and LPS-induced lethal shock in systemic inflammation
Source: Front Immunol. 2025 Oct 23;16:1610033. doi: 10.3389/fimmu.2025.1610033 (PMC12589025; doi:10.3389/fimmu.2025.1610033)

Supplemental Figure 6

Histogram showing total leukocyte, neutrophil, eosinophil and monocyte/macrophage counts in BALF, and leukocyte counts in peripheral blood. Cells were counted using a Hematometer Multispecies Hematologic Analyzer. Data n=4-6 per group from 2 independent experiments. Con, vehicle controls.*p < 0.05, **p<0.01, ***p<0.001, ****p<10^{-4}.

Supplemental figure 6

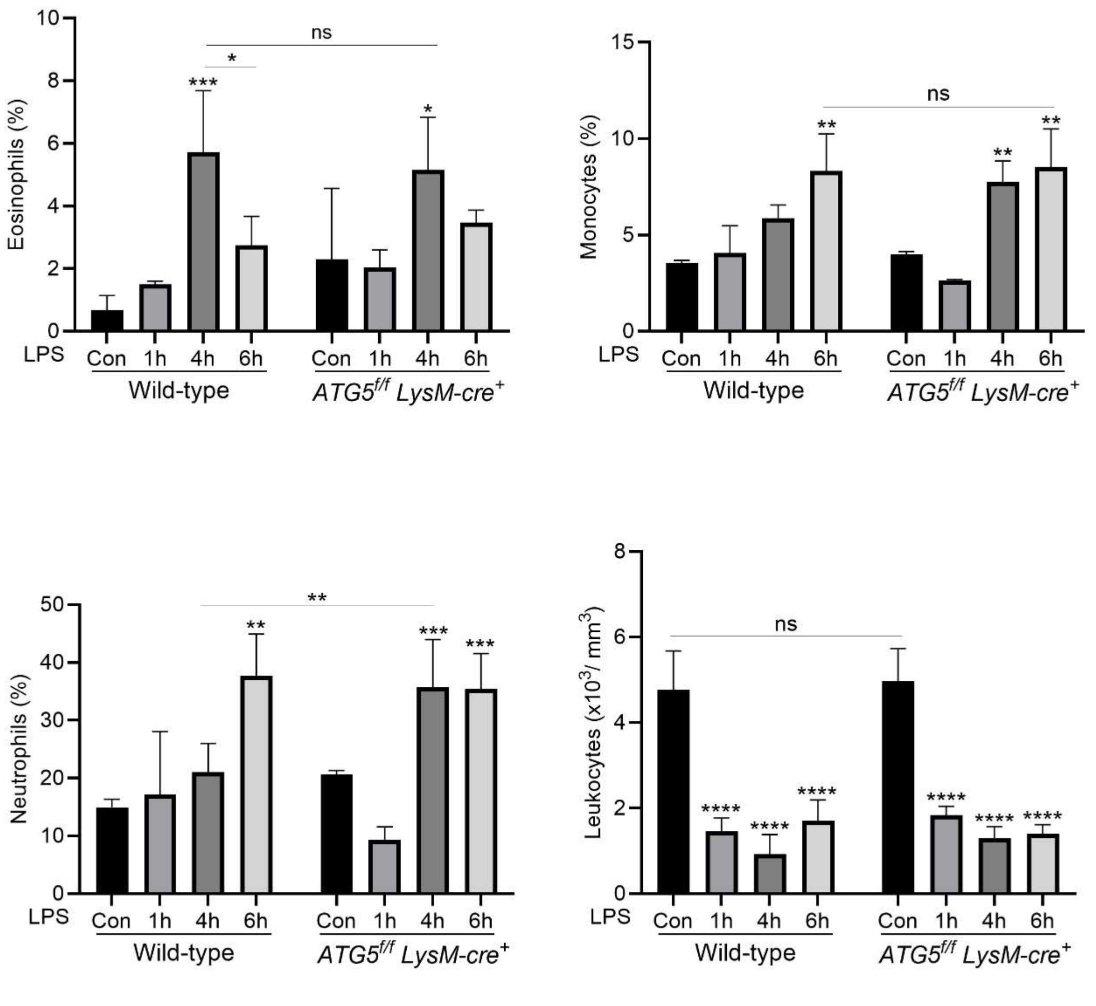

Supplement: Supplementary file 6 [file DataSheet6.pdf]
