# Supplementary material for: Disrupted macrophage autophagy as a driver of cell death and LPS-induced lethal shock in systemic inflammation
Source: Front Immunol. 2025 Oct 23;16:1610033. doi: 10.3389/fimmu.2025.1610033 (PMC12589025; doi:10.3389/fimmu.2025.1610033)

Supplemental Figure 7

Gating strategy of neutrophils, eosinophils and macrophages in the liver. Cells isolated from the liver of LPS stimulated (6 hours) *Atg5*$^{f/f}$*LysM-cre*$^{+}$ and wild-type mice and vehicle controls, were analyzed by flow cytometry. Following FSC-SSC singlet gating and exclusion of dead cells (L/D), pre-gated CD45 positive cells were further gated for the subsets of interest. Left panels show gating of CD45^{+} SiglecF^{-} Ly6G^{+} CD11b^{+} neutrophils; Middle and right panels shows CD45^{+} F4/80^{+} macrophages, and CD11b^{low}, CD206^{+} and Nos^{+} subsets.

Supplemental figure 7

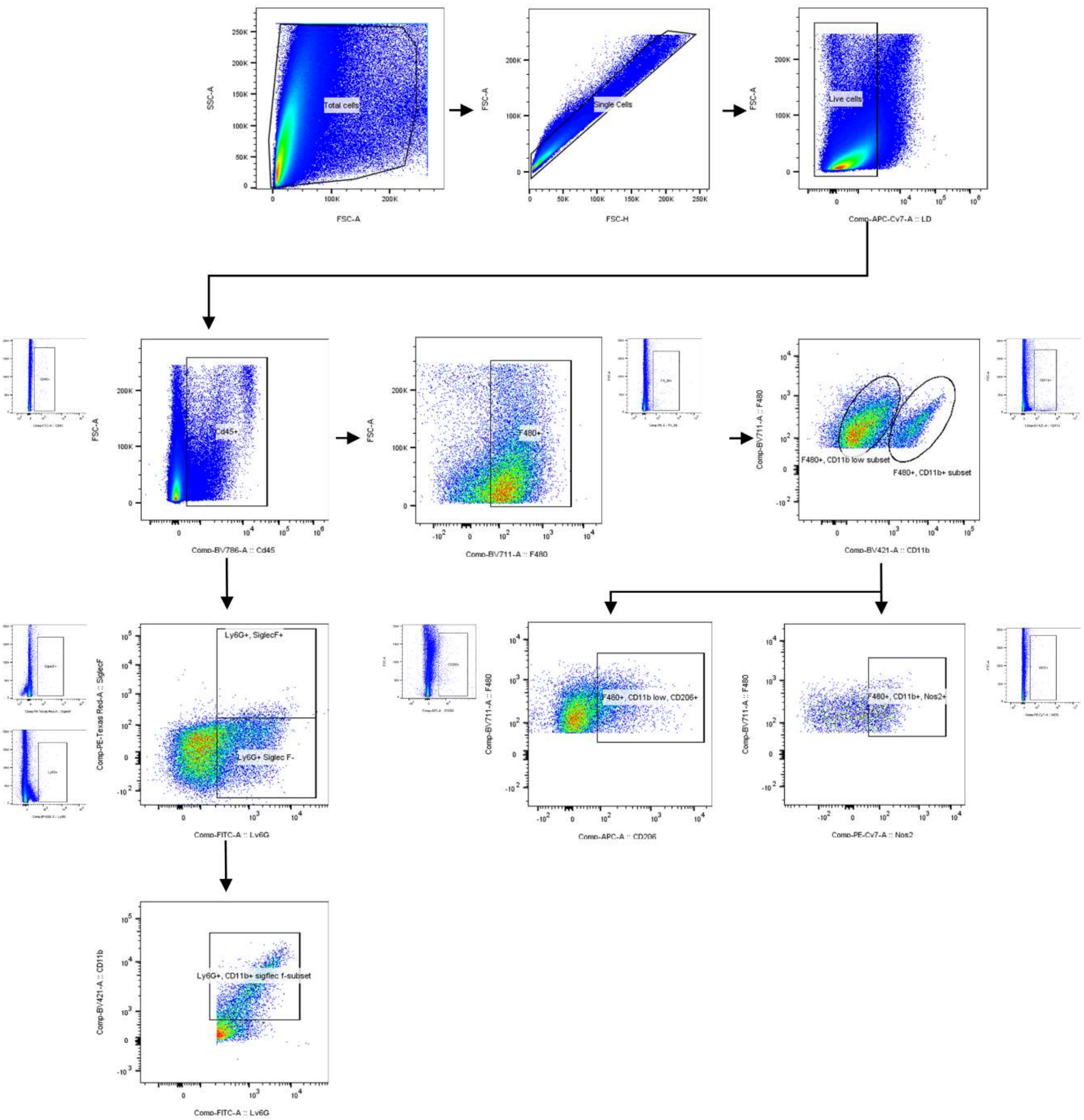

Supplement: Supplementary file 7 [file DataSheet7.pdf]
