# Supplementary material for: Disrupted macrophage autophagy as a driver of cell death and LPS-induced lethal shock in systemic inflammation
Source: Front Immunol. 2025 Oct 23;16:1610033. doi: 10.3389/fimmu.2025.1610033 (PMC12589025; doi:10.3389/fimmu.2025.1610033)

Supplemental Figure 8

A, Detection of Nos2 and CD206 by immunofluorescence in BMDMs. BMDMs were incubated with LPS (100 ng/mL) and cells were analyzed after 1, 4, 24, 48 and 72 hours of incubation. Representative images of immunofluorescence staining of BMDMs for Nos2 (red), CD206 (green), and nuclei stained with DAPI (blue). Zeiss Observer Z7 fluorescence microcopy imaging; original magnification 20x. The representative images shown are from 2 independent experiments. Histograms on right were quantification of mean fluorescence intensity per cell (MFI) and total cell number using ImageJ in randomly selected fields and averaged. **B**, Histogram of cytokine levels in medium of BMDMs incubated with LPS. ELISA results for inflammatory cytokine Tnfα and Il-1β production in cell culture medium. Data (n=4 per group) and presented as the mean ± S.D. **C**, Histogram of markers for macrophage polarization in BMDMs incubated with ferric ammonium citrate (100 µM, 16 hours). Quantitative PCR of *Retlna*, *Il-10*, *Mrc1*, *Tnf*α mRNA in BMDMs. Con, vehicle controls. $*p<0.05$; $**p<0.01$; $***p<0.001$; $****p<10^{-4}$.

Supplemental figure 7

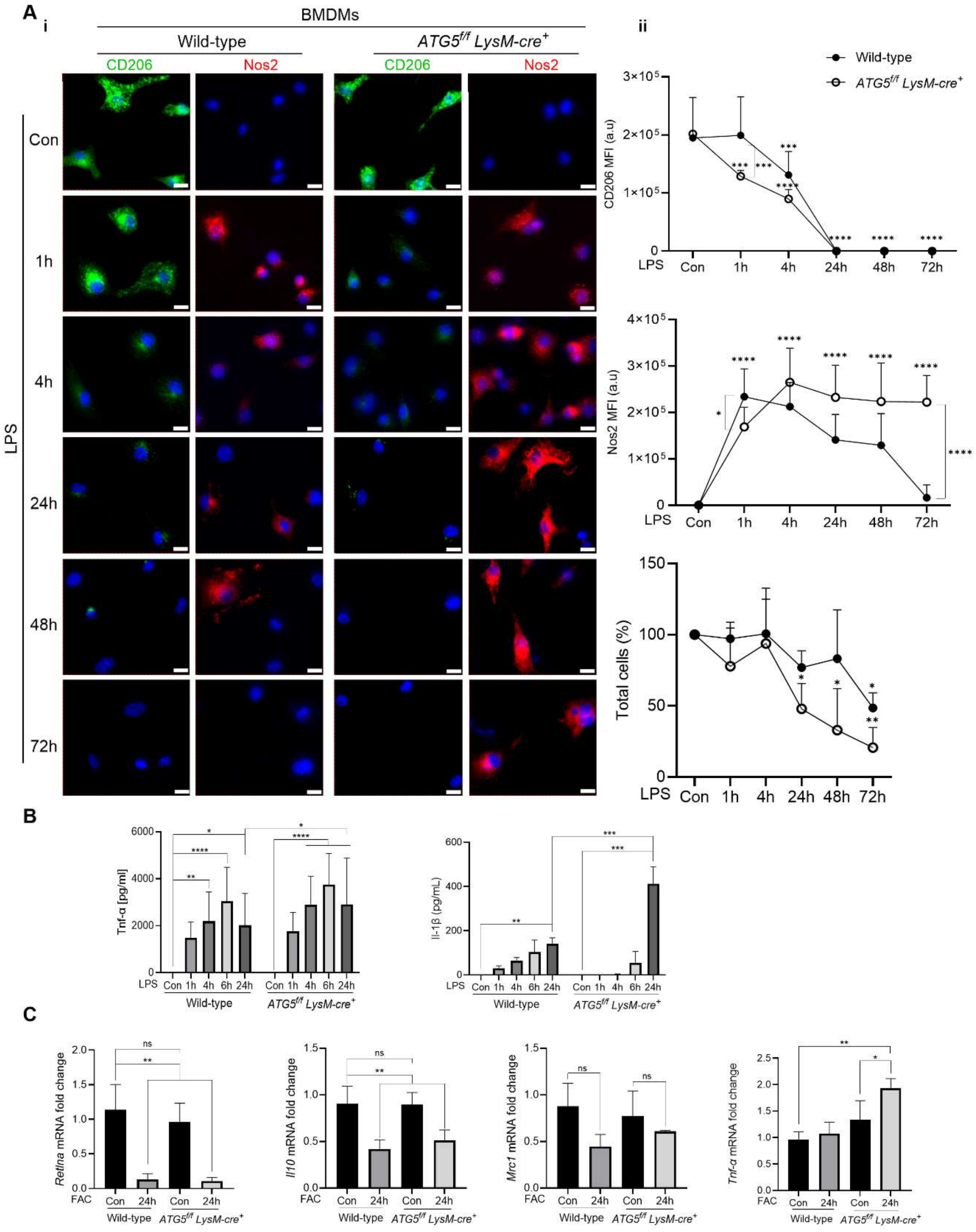

Supplement: Supplementary file 8 [file DataSheet8.pdf]
