# Supplementary material for: Disrupted macrophage autophagy as a driver of cell death and LPS-induced lethal shock in systemic inflammation
Source: Front Immunol. 2025 Oct 23;16:1610033. doi: 10.3389/fimmu.2025.1610033 (PMC12589025; doi:10.3389/fimmu.2025.1610033)
Supplement: Supplementary file 9 [file DataSheet9.pdf]

Supplemental table 1

Antibodies used for flow cytometry, immunofluorescence (IF), and western blot (WB).

Source/Isotype	Antibody		Ref
Rabbit	Lc3b (550 µg/mL); (WB:1/1000)	Proteintech	14600-1-AP
Rabbit	Caspase-1 (E9R2D) (100µL; WB:1/1000)	Cell signaling	83383s
Rabbit	Caspase-3 (ASP175) (100 µL; WB:1/1000)	Cell signaling	9661s
Mouse	β-actine (200µl) (WB:1/2000)	Sigma	A3854
Rabbit	Il1-b (D4T2D) (100 µL; WB:1/1000)	Cell signaling	12426s
Rabbit	p62 (SQSTM1) (200 µL; WB:1/1000)	Sigma	p0067
Rabbit	Nlrp3 (D4D8T) (100 µL; WB:1/1000)	Cell signaling	15101s
Rabbit	Caspase-8 (D35G2) (100 µL; WB:1/1000)	Cell signaling	4790s
Mouse	Caspase-3 cl (100 µg; WB:1/500)	Euromedex	ARG66888
Rabbit	Caspase-8 cl (Asp387) (D5B2) (100 µL;WB:1/500)	Cell signaling	8592s
Mouse	Caspase-9 (C9) (100 µL; WB:1/1000)	Cell signaling	9508s
Rabbit	Cleaved GsdmD(Asp276) (E3E3P) (100 µL;WB:1/500)	Cell signaling	10137s
Rabbit	GsdmD(E9S1X) (100µL;WB:1/1000)	Cell signaling	39754
Rabbit	Il-18(E8P5O) (100 µL;WB:1/1000)	Cell signaling	57058s
Rabbit	APG5L/ATG5 [EPR1755(2)](100 µl;IF:1/100)	Abcam	108327
Rabbit	Mannose Receptor (1 mg/mL;IF:1/100)	Abcam	ab64693
Goat	Anti-Rabbit IgG (H+L) Cross-Adsorbed Secondary Antibody, Alexa Fluor™ 488(1 mg/mL)(IF:1/800)	Invitrogen	A-11008
Goat	Anti-Rabbit IgG (H+L) Cross-Adsorbed Secondary Antibody, Alexa Fluor™ 594 (1 mg/mL);(IF:1/800)	Invitrogen	A-11012
Rabbit	Mouse Metal Transporter Protein 1/Ferroportin	Alpha diagnostic intl	MTP11-S

	(MTP1/IREG1/Fpn) antiserum<br>(1 mg/mL) (IF:1/100, WB:1/1000)		
Mouse	GPX4 (3F5G5)<br>(150 µL; WB:1/1000)	Proteintech	67763-1-Ig
Rabbit	Transferrin<br>(WB:1/1000)	GeneTex	GTX32933
Rabbit	Haptoglobine<br>(WB:1/1000)	GeneTex	GTX55653
Rabbit	Ferritin H (SC0620)<br>(100 µL; WB:1/1000)	Invitrogen	MA5-32244
Rabbit	Human/Mouse<br>Myeloperoxidase/MPO<br>(0.2 mg/mL);(1/200)	R D systems	AF3667
Mouse	CD68(100 µg)(IF:1/100)	Enquirebio	968-MSM2-P1
Goat	Anti-rabbit IgG, HRP-linked antibody (1 mL; WB:1/2000)	Cell signaling	7074s
Horse	Anti-mouse IgG, HRP-linked antibody (1 mL;WB:1/2000)	Cell signaling	7076s
Rat	Anti mouse CD16/CD32 clone 93<br>(1 mg/mL) FACS:1/200	Invitrogen	16-016185
Rat	PE CF594 Anti Mouse Siglec-F Clone E50-2440<br>(0.2 mg/mL) FACS:1/200	BD science	562757
Rat	FITC anti mouse Ly-6G clone 1A8<br>(0.5 mg/mL) FACS:1/200	BioLegend	127606
Rat	BV785 anti mouse CD45 clone 30-F11<br>(0.2mg/mL) FACS:1/200	BioLegend	103149
Rat	BV 711 anti mouse F4/80 clone T45-2342<br>(0.2 ml/mL) FACS:1/300	BD science	565612
Rat	BV421 anti mouse/humain CD11b clone M1/70<br>(0.2 mg/mL) FACS:1/200	BioLegend	101251
Rat	Cyanine 7 anti mouse iNOS PE clone CXNFT<br>(0.2 mg/mL) FACS: 1/200	Invitrogen	25-5920-82
