# Supplementary material for: Disrupted macrophage autophagy as a driver of cell death and LPS-induced lethal shock in systemic inflammation
Source: Front Immunol. 2025 Oct 23;16:1610033. doi: 10.3389/fimmu.2025.1610033 (PMC12589025; doi:10.3389/fimmu.2025.1610033)
Supplement: Supplementary file 10 [file DataSheet10.pdf]

Supplemental Table 2

Primers of hepcidin (*Hamp1*) used for qPCR.

Hamp1 forward	5’-CCTATCTCCATCAACAGATG-3’
Hamp1 reverse	5’-TGCAACAGATACCACACTG-3’
