# Supplementary material for: Disrupted macrophage autophagy as a driver of cell death and LPS-induced lethal shock in systemic inflammation
Source: Front Immunol. 2025 Oct 23;16:1610033. doi: 10.3389/fimmu.2025.1610033 (PMC12589025; doi:10.3389/fimmu.2025.1610033)
Supplement: Supplementary file 11 [file DataSheet11.pdf]

Supplemental Table 3

Score for grading histological severity of liver damage

Score	Congestion
0	None
1	Slight
2	Mild
3	Moderate
4	Severe

Score	Sinusoidal dilatation
0	Normal sinusoids (no enlargement)
1	Mild enlargement ; < 25% of the sinusoids in the field affected
2	Moderate enlargement 25–50% of the sinusoids in the field affected
3	Severe enlargement > 50% of the sinusoids in the field affected

Score	Cell infiltration
0	Normal infiltration (control level)
1	Mild infiltration (120–200 %)
2	Moderate infiltration (200–300 %)
3	Marked infiltration (300–400 %)
4	Severe infiltration (> 400 %)

Score for grading histological severity of spleen damage

Score	Eosin-stained area	
0	absent	absent – no
1	mild	focal, rare areas <10% of red pulp
2	moderate	multiple areas, 10–30% of red pulp
3	marked	diffuse areas, >30% of red pulp
4	severe	almost all red pulp replaced

Score	Spleen weight/mouse weight	
0	absent/normal	≤ 0.2%
1	mild	0.21–0.4%
2	moderate	0.41–0.8%
3	marked	0.81–1.2%
4	severe	> 1.2%

Score	Number of red pulp cells	
0	absent/normal	Within control range (mean ± 1 SD).
1	mild	± 25–50% (1 and 2 SD)
2	moderate	± 51–100%. (2 and 3 SD)
3	marked	±101–200%. (3 and 4 SD)
4	severe	> 200% of control value. (> 4 SD)
